# Deep learning-assisted mapping of dendritic spines using sequential 2D two-photon calcium imaging

**DOI:** 10.1016/j.isci.2026.117010

**Published:** 2026-08-01

**Authors:** Dario Cupolillo, Vincenzo Regio, Valentina Galleano, Andrea Barberis

**Affiliations:** 1 Istituto Italiano di Tecnologia, Synaptic Plasticity of Inhibitory Networks, 16163 Genova, Italy; 2 The Open University, School of Life, Health and Chemical Sciences, Milton Keynes MK7 6AA, UK

**Keywords:** dendritic spines, synaptic spatial organization, sequential dendritic imaging, two-dimensional two-photon microscopy, calcium imaging, single-spine calcium imaging, calcium event classification

## Abstract

Neurons transform complex spatiotemporal synaptic inputs into structured action potential sequences. Excitatory inputs initiate or interact with dendritic spikes or plateau potentials, adding computational layers that diversify input-output transformations. Because synapse location strategically shapes the dendritic events, mapping synaptic organization is critical for understanding neuronal function. Spine calcium imaging offers a direct readout of active contact location but requires access to spines distributed across intricate three-dimensional dendrites. We present a software pipeline for targeted dendritic imaging and analysis using sequential two-dimensional scanning on standard two-photon microscopes. It includes a ScanImage-compatible pre-acquisition tool, ROIpy, which generates dendrite-aligned region-of-interests (ROIs) for depth-specific dendritic imaging, and a post-acquisition suite, Spyne, which includes deep learning modules for spine detection and binary classification of calcium activity (active vs. non-active spines). This method accommodates diverse experimental designs, including two-photon imaging with patch-clamp or all-optical setups, supporting whole-arbor or branch-specific imaging.

## Highlights

• ROIpy converts 3D reconstructions into 2D ScanImage-compatible dendritic ROIs
• Spyne segments dendritic spines using three deep learning backends
• A custom CNN classifier detects calcium event presence with high accuracy
• The pipeline maps active synaptic inputs to dendritic branches

## Graphical abstract

**Figure:**
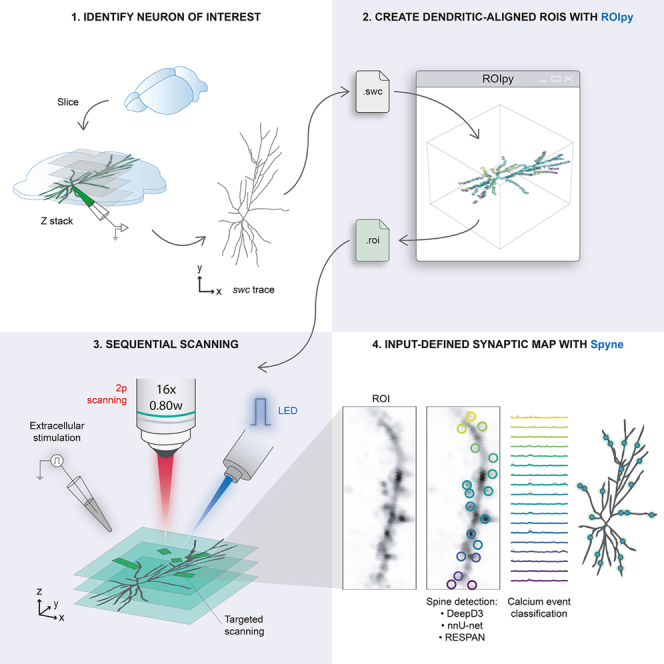

## Teaser

Applied sciences; Software engineering

## Introduction

The complexity of cognitive functions reflects the capacity of individual neurons to process a multitude of information and return appropriate responses.^1^^,^^2^ Neurons continuously transform arrays of inputs received onto thousands of dendritic spines into meaningful neural codes in the form of action potential patterns. The variety of neuronal input-output (I-O) transformation is enhanced by stacking layers of non-linear integration. This model holds that dendrites locally process inputs as an independent unit and feed the output to layers below.^3^^,^^4^^,^^5^ For instance, in pyramidal neurons (PNs), large depolarizations in dendritic compartments produce local regenerative events such as dendritic spikes (d-spikes) through the activation of voltage-dependent Na^+^ channels^6^ or slow plateau potentials through voltage-gated Ca^2+^ channels (VGCCs)^7^ and N-methyl-D-aspartate receptors (NMDAr).^8^^,^^9^^,^^10^^,^^11^ In combination with the distribution of active conductances, the spatial organization of inputs throughout the dendritic tree influences such local responses.^12^ In PNs, clusters of co-active spines can trigger the generation of fast, low-traveling d-spikes (like Na^+^ spikes), producing supralinear summation of inputs and enhancing coincidence detection.^6^^,^^13^^,^^14^^,^^15^ On the other hand, long-lasting plateau-like events mediated by NMDAr and VGCC support the integration of spatially or temporally dispersed input sequences across behavioral timescales.^16^^,^^17^ Understanding dendritic integration within defined temporal windows requires dissecting the temporal and spatial arrangement of synaptic inputs, their identity, and their interaction. Two-photon glutamate uncaging has revealed key mechanisms of non-linear dendritic processing and synaptic interaction,^6^^,^^18^^,^^19^ but it lacks information on presynaptic identity, relies on artificial input patterns, and is typically restricted to subportions of dendritic arbors. Anatomical labeling approaches such as GRASP (GFP reconstitution across synaptic partners) can effectively map the whole-cell distribution of identified synaptic inputs^20^^,^^21^^,^^22^ but do not capture the temporal and functional dynamics. Calcium imaging, by contrast, provides a direct readout of synaptic activation. When combined with optogenetics, it enables the inference of input identity, for example, through activation of opsins specifically expressed in defined subpopulations. For spine-level calcium imaging, laser scanning confocal or multiphoton microscopy assessing Ca^2+^-sensitive GECIs (genetically encoded calcium indicators, e.g., green calcium-modulated protein GCaMP6s) or synthetic dyes (e.g., Fluo-4FF, Fluo-5F, Fura-2, and OGB-1) remains the method of choice for both *ex vivo* and *in vivo* settings.^23^^,^^24^^,^^25^^,^^26^^,^^27^^,^^28^ Laser scanning strategies must balance temporal resolution, spatial coverage, and spatial resolution. For instance, galvo-based systems can perform targeted scanning within spatially confined regions to increase the frame rate and resolution.^29^ Nevertheless, sequential acquisition of two-dimensional (2D) regions of interest (ROIs) across multiple *z* planes can fully capture the three-dimensional (3D) topology of dendritic arbors, generating a volume of discrete z layers with sparse temporal resolution. Here, we describe a modular pipeline for mapping dendritic spines activated by the selected inputs, using sequential 2D two-photon scanning microscopy. The pipeline is built on two main packages: ROIpy and Spyne. ROIpy is a pre-acquisition tool for generating 2D ROIs aligned with dendritic trajectories, which integrate with scattered scanning featured by the popular data acquisition software ScanImage.^30^ Spyne is a post-acquisition data analysis tool, which includes automatic identification of dendritic spines using deep learning-based segmentation methods, including DeepD3,^31^ nnU-Net,^32^ and the RESPAN pipeline,^33^ followed by custom spine calcium event detection. For the latter, we propose a deep learning-based classifier to detect the presence of a calcium spike across thousands of spines. In this work, we report on the pipeline in its most comprehensive experimental setting, combining full dendritic calcium imaging with simultaneous patch-clamp recordings. Nonetheless, the approach offers high flexibility: it extends to all-optical configurations, combining GECIs and optogenetics, and can be tailored to target specific dendritic compartments or smaller tree subregions based on experimental needs. Overall, the approach enables efficient, high-throughput analysis of dendritic inputs across broad arbor segments without requiring volumetric scanning.

## Results

### ROIpy produces rectangular ROIs aligned with dendritic trajectories

ROIpy converts 3D dendritic reconstructions into sets of small, scattered 2D ROIs, each capturing a short dendritic segment at a discrete focal depth, enabling targeted sequential imaging of extended arbors on standard two-photon systems (Figures 1A–1C). To generate the structural reconstruction required for ROI generation, we patched ventral hippocampal CA1 PNs (vCA1 PNs) in acute brain slices obtained from adult mice and filling them with Alexa 594 (A594) and Fluo-5F through the patch pipette to visualize dendritic morphology and monitor calcium activity, respectively (Figure 1A). All data were collected from 14 vCA1 PNs across 14 mice. Following dye loading, we acquired a z stack of the whole dendritic arborization (z step: 1–1.5 μm). We used this stack as reference to reconstruct the 3D morphology of the filled neuron (Figure 1B) through the simple neurite tracer (SNT)^34^ plugin of ImageJ-Fiji,^35^ producing a standard SWC file.^36^ This format encodes neurons as a set of interconnected nodes with *x*, *y*, and *z* coordinates, parent-child connectivity, radius, and compartment identity (Figure 1B). ROIpy parses these nodes to generate layer-grouped rectangular ROIs (Figures 1C and 1D), storing them as json-formatted ROI files—one per imaging plane—compatible with ScanImage’s multiple ROI (mROI) imaging tool. ROI generation is rapid, completing in a few seconds and scaling linearly with the reconstructed arbor size (Figures S1A–S1C). ROI generation is a multi-step process, which includes initial ROI placement, shape adjustment, and pixel allocation (see STAR Methods and Table S1). Shortly, nodes are first grouped by *z* plane and then by uninterrupted sequences with direct parent-child connectivity, yielding discrete dendritic segments that define initial ROI placement and orientation. ROIs are then post-processed to achieve final configuration. Short rectangles that are wider than their length are merged with adjacent ROIs or discarded (Figures S2B–S2D). ROIs capturing curving dendrites are extended along the width axis to ensure coverage. Then, all ROIs are uniformly stretched along their major axis by a fraction of their height (see STAR Methods). ROIs whose spatial overlap exceeds a defined threshold are removed to avoid redundant scanning (Figure S2D). A graphical user interface supports semi-automated ROI placement and adjustment (Figure S3A). During acquisition, ROI groups are scanned sequentially. All ROIs assigned to a given imaging plane (*Z*_*n*_) are scanned in a single frame, after which the focal plane is manually stepped to the *Z*_*n+1*_, and the corresponding ROI set is acquired (Figure 1E). Repeating this process across successive planes yields a volumetric dataset composed of dendritic-aligned ROIs sampled throughout the arbor at discrete z layers (Figure 1F). Overall, the 2D projection of all ROIs covered 3.39% ± 0.54% of the entire field of view (Figure S4C). The final distribution and shape of ROIs per plane depend ultimately on the density of dendritic segments crossing them, usually with dense packing near the soma and sparse coverage at distal planes. The total scan area, thus, differs substantially across z layers. To maintain a constant frame rate regardless of scan area, and thereby ensure sufficient temporal resolution for detecting fast spine calcium transients at every depth, pixel dimensions were adjusted independently per z layer (Figure 1G; see STAR Methods for details). We then evaluated ROI placement accuracy. ROIs enclosed 81.61% ± 1.90% of SWC nodes, indicating effective sampling of the reconstructed arbor (Figures S4A and S4B). Intensity profiles along scan lines orthogonal to the dendritic shaft showed a peak centered at the ROI midline with a full width at half maximum (FWHM) of 1.92 ± 0.12 μm, consistent with the expected diameter of dendritic segments and the optical resolution of the system (Figures S4E and S4F). Finally, we computed a peak-to-background ratio (ratio of ROI-center/ROI-periphery intensity) of 87.71 ± 19.26, indicating that fluorescence at the ROI center was on average ∼88-fold higher than that at the periphery (Figure S4G). Beyond ROI generation, ROIpy includes a suite of morphological visualization and basic structural analysis. To showcase the broad applicability of ROIpy across cell types and preparations, we analyzed a neuronal morphology dataset of 63 fully reconstructed dendritic arborizations from layer-5 (L5) PNs of primary visual areas (VISp) obtained from the Allen Cell-Types Database^37^^,^^38^ (Figure S5A) (summarized in Table S2). ROIpy resolved individual branches—defined as segments between a soma or branch point and a terminal node—and sorted them by the branching order and compartment type (Figure S5A). We calculated a mean total dendritic length of 4.46 ± 0.17 mm across 47.9 ± 2.0 branches on average (Figure S5B). Sholl analysis^39^ revealed a branching profile consistent with L5 PNs,^40^ characterized by dense limited basal arborization within 200 μm from the soma and oblique apical dendrites stemming from a main trunk (elongating for up to 400 μm) that, in turn, furcates at the tuft beyond 400 μm (Figure S5C). Convex hull analysis of terminal nodes yielded a mean dendritic volume of 0.0085 ± 0.0006 μm^3^ (Figure S5D).

**Figure 1:**
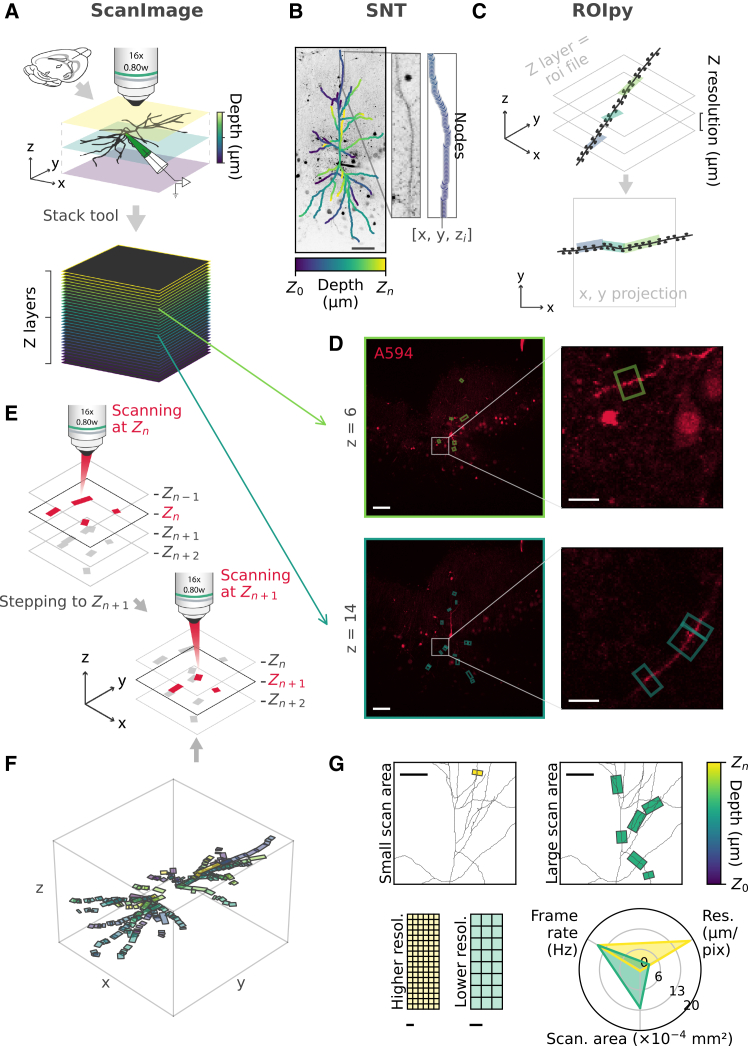
Automated targeted multi-ROI scanning (A) Schematic of the first step of the pipeline. Top: a vCA1 neuron in horizontal acute slices is patched under 2-photon microscope. Bottom: a multi-layer z stack of the filled arbor is acquired using the ScanImage stack acquisition tool. (B) Neuronal morphology reconstruction. A three-dimensional SWC reconstruction is generated from the z stack, using simple neurite tracer (SNT) in Fiji. Left: representative maximum projection of the A594-filled neuron with reconstruction overlayed. Middle: zoomed view of an apical dendritic segment. Right: corresponding node sequence, with nodes defined by *x*, *y*, and *z* coordinates. Scale bar: 50 μm. (C) ROI generation. ROIpy uses the morphological reconstruction to place two-dimensional rectangular ROIs aligned with dendritic trajectories across focal depths. Top: schematic of ROIs distributed across individual *z* planes, with each plane corresponding to one ROI file. Bottom: *xy* projection illustrating ROIs following a representative dendritic branch. (D) Representative single-layer images from the neuron shown in (B) at two focal depths (top, layer 6; bottom, layer 14). ROIs are overlaid and color coded by depth. Insets show a zoomed view of a *xy* region at each depth, illustrating ROI alignment with local dendritic segments. Scale bars: 50 μm: inset scale bars: 10 μm. (E) Sequential acquisition strategy. Top: all ROIs assigned to a given *z* plane (*Z*_*n*_) are loaded into the ScanImage mROI module and imaged within a single frame. Bottom: the focal plane is then stepped to *Z*_*n+1*_, and the corresponding ROI group is acquired. The process is repeated across all layers. (F) Representative volume projection of all ROIs, color coded by depth. Box dimensions (*xyz*): 280 × 280 × 466 μm. See also Figure S2. (G) Per-layer adaptation of spatial sampling. Top: two representative z layers from the same neuron with different ROI densities. ROIs are overlaid on the *xy*-projected morphology (gray) and color coded by depth. Planes containing a few ROIs (left) have smaller total scan areas; planes containing many ROIs (right) have larger total scan areas. Scale bars: 25 μm. Bottom: pixel dimensions are adjusted per layer to maintain a constant frame rate regardless of scan area. Planes with small scan areas are sampled at higher spatial resolution; planes with large scan areas are sampled at lower resolution. Scale bars: 1 pixel. The radar plot summarizes the trade-off between the scan area, spatial resolution, and frame rate, with acquisition held constant at 16 Hz across all layers.

### Mapping dendritic spines from discrete 2D ROIs

To map dendritic spines and identify those targeted by a defined axonal input, we used stimulus-evoked calcium transients within spine heads as a readout of active synaptic contacts. Stimulation was delivered either electrically or optogenetically. For electrical stimulation, we placed an electrode in the *stratum radiatum* of the ventral hippocampal slices to stimulate the Schaffer collateral (SC) bundle originating from vCA3. For optogenetics stimulation, we injected CaMKIIα-ChR2-EYFP into the basolateral amygdala (BLA) to stimulate ChR2-expressing projection terminals within vCA1^41^ (Figure 2C). During scanning, we delivered either five electrical paired pulses (PPs) at 0.1 Hz or five single blue light pulses (470 nm, 2 ms) at 0.1 Hz, both evoking subthreshold excitatory postsynaptic currents (EPSCs) in vCA1 PNs (Figure 2C). To prevent detector saturation and photodamage associated with concurrent imaging and optogenetic, we used gated photomultiplier tubes (PMTs), which allow millisecond-timescale electrical gating of signal collection. This stimulation protocol was repeated for every z layer, recording A594 and Fluo-5F signals from ROIpy-generated ROIs across the full arbor. Raw data were processed offline using Spyne, a custom analysis suite for image inspection, gating correction, spine segmentation, and calcium event detection. Raw data were unpacked into 2D frame sequences per ROI and pre-processed by 3D median filtering and correction of gating artifacts as needed (Figure S6). Pre-processing completed in a few minutes, scaling with the dataset size (Figures S1D and S1E). A reference image for each ROI was generated by computing the maximum intensity projection of the A594 signal across all frames and trials, revealing a high-contrast delineation of dendrites and spine profiles (Figures 2A and 2B). Spine segmentation was performed using three deep learning backends—DeepD3,^31^ nnU-net,^32^ and RESPAN^33^ (nnU-net that incorporates content-aware image restoration, CARE)^42^—each trained and optimized on our own dataset (see STAR Methods and Table S3). The CSBDeep restoration model was trained on the same dataset as the segmentation network, with low-signal-to-noise ratio images generated synthetically through controlled image degradation^42^ (Figure S8; see STAR Methods; Table S4). Each backend returns binary spine masks (Figure 2B) that are further processed to yield individual spine instances (Figure S7D; see STAR Methods). To benchmark segmentation performance, all three models were evaluated against manual annotations from five independent human raters across 5 dendritic ROIs, using centroid-based matching.^31^ DeepD3, nnU-Net, and RESPAN achieved mean recalls of 0.88, 0.89, and 0.88, respectively, compared with a mean inter-rater recall of 0.76, demonstrating that all three models perform at or above the level of human inter-rater agreement (Figures S7E–S7G). DeepD3 inference was substantially faster than nnU-Net, with more favorable scaling as a function of spine count (Figure S1F). We re-mapped spine centroids into the full field-of-view coordinate space, using affine matrix transformation, obtaining a whole-arbor spine position map. Spine positions in this map are represented as 2D centroids within their respective discrete acquisition planes (Figure 2E). We extracted the Fluo-5F signal encased by spine masks across time (Figure 2D) and computed Δ*F*/*F*_0_ by normalizing background-corrected pixel values to a rolling percentile baseline (see STAR Methods; Equation 5). We further denoised the time series (t-series) by applying a modified Okada filter^23^ and *Z* scored to standardize the signal amplitude across neurons and trials (Equation 6). Because the sequential acquisition protocol entails repeated stimulation and imaging across all z layers, we assessed whether cumulative exposure induced photobleaching or altered spine responsiveness. Normalized ROI pixel intensity remained stable across z depths (Figure S9B). Comparably, the mean spine density did not differ significantly across z layers (Figure S9C), and the fraction of responsive spines responding was uniform with depth (Figures S9D and S9E). These data indicate that repeated imaging did not induce detectable photobleaching or systematically alter spine activity across the acquisition volume.

**Figure 2:**
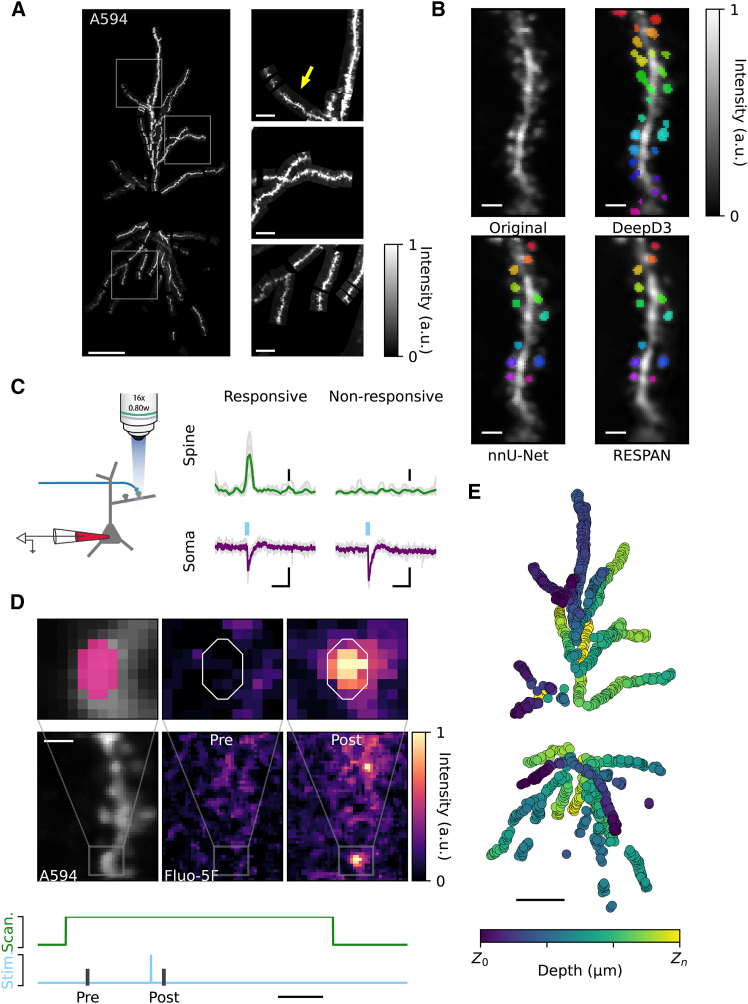
Spine semantic segmentation and calcium imaging (A) Structural imaging of the dendritic arbor from ROI acquisitions Left: *xy* projection of all acquired ROIs in their original field-of-view positions. Boxes indicate regions shown at higher magnification on the right. Scale bar: 50 μm. Right: three representative dendritic regions reconstructed from maximum intensity projections across frame sequences within individual ROIs. Yellow arrow indicates the ROI shown in (B). Scale bars: 10 μm. (B) Representative spine segmentation masks obtained with three automated segmentation backends (DeepD3, nnU-Net, and RESPAN). Scale bars: 2 μm. See also Figures S7 and S8. (C) Stimulation and imaging configuration. Left: schematic of a patched vCA1 neuron receiving optogenetic stimulation via 470 nm light, activating channel rhodopsin-expressing BLA afferent terminals. Right: representative Fluo-5F traces from a responsive and a non-responsive spine (top) and simultaneously recorded somatic EPSCs (bottom), temporally aligned to the stimulation onset. Light blue bar indicates light stimulation onset. Scale bars: 0.5 Δ*F*/*F*_0_; 10 pA; 500 ms. See also Figure S6. (D) Single-trial calcium response in a representative responsive spine. Top: A594 reference image with the spine of interest indicated in pink (left); Fluo-5F frame acquired immediately before (center) and after (right) optogenetic stimulation, showing stimulus-evoked fluorescence increase. Scale bar: 2 μm. Bottom: data acquisition (DAQ) board triggers waveform for optogenetic stimulation (light blue) aligned with the two-photon acquisition window (green). Scale bar: 500 ms. (E) Spatial map of detected spine centroids across the full field of view based on DeepD3 segmentation. Each point represents the centroid position of one spine, color coded by focal depth.

### Custom deep learning classifier automatically identifies active spines

Manual detection of calcium events across thousands of spines is labor intensive and prone to errors and inconsistencies. To streamline and automate this step, we trained a supervised binary classifier to detect stimulus-evoked calcium events in discrete, univariate Δ*F*/*F*_0_ t-series. The ground-truth training dataset comprised 2,204 expert-annotated traces, labeled as events (1) or no-event (0) in near-equal class proportions (no-event: *n* = 1,154, 52.4%; event: *n* = 1,050, 47.6%). Data were stratified and split into a training set and a validation set (in 0.75/0.25 ratio). The classifier architecture consists of three residual convolutional blocks followed by a max-pooling layer and a fully connected output (Figure 3A; Table S5) typically used for t-series classification.^43^ During training, we monitored the F1 score (Equation 9). The model converged within 80 epochs, with training and validation loss functions decreasing at comparable rates (Figure 3B). The F1 score showed a steep initial rise—reflecting rapid gains in the balance between precision (Equation 10) and recall (Equation 11)—followed by a plateau indicating stable convergence (Figure 3C). On an independent never-seen-before test dataset of 459 annotated Δ*F*/*F*_0_ t-series (no-event: *n* = 245; event: *n* = 214), the model achieved an area under the precision-recall (PR) curve of 0.98 (Figure 3D). When ranked by the probability of containing a calcium event, predictions closely mirrored human scoring (Figure 3E). Applying a decision threshold selected to maximize F1 score on the test set (Figure S10A), the classifier achieved a precision of 93.9% and 93.2% and recall of 93.9% and 96.7% for no-event and event classes, respectively, reflecting reliable and high-sensitivity classification for both classes (Figure S10C). Overall classification accuracy was 95.2%, with a mean detection runtime of a few milliseconds per trace (Figure S1G). To examine the internal representation learned by the network, we extracted activations from the final hidden layer for all samples and projected them into a 2D space, using uniform manifold approximation and projection (UMAP).^44^ The resulting embedding revealed consistent segregation of traces across datasets (Figure S10D), indicating that the network learned a generalizable latent representation. The inter-class to intra-class distance was 2.98 for training and validation sets and 2.48 for the test set. Silhouette scores were 0.54 and 0.46 for the combined training/validation set and test set, respectively, with the modest reduction in the test set possibly reflecting its smaller sample size. We next benchmarked the classifier against human annotations and classical detection methods. Five independent raters annotated the held-out test dataset. Inter-rater recall ranged from 0.61 to 1.00 (mean 0.82), reflecting natural variability in human annotation. The model’s recall against each individual rater achieved 0.91–0.97 (mean 0.94), matching or exceeding the average inter-rater agreement (Figure S10E). We tested cut-off values of 1.96 and 2.5 times the standard deviation (*σ*) of the baseline (Figure S10F). The 1.96*σ* threshold achieved an accuracy of 82% with high event sensitivity of 94.3% but limited specificity (71.4%), indicating an elevated false-positive rate (Figure S10G). Raising the threshold to 2.5*σ* improved the overall accuracy to 85.1%, with sensitivity of 93.8% and specificity of 77.6% (Figure S10H). Finally, we fit the labeled training dataset to a logistic function (Equation 12), which separated the test dataset based on the dot product of weights and Δ*F*/*F*_0_ features (Figure S10I). The regression model displayed an area under the receiver operating characteristics (ROC) curve of 0.96 (Figure S10J). After optimizing the threshold to maximize the F1 score, the regression model yielded recall of 94.8% for events and 86.9% for no-events, and event precision of 86.2%, at an overall accuracy of 90.6% (Figure S10K). Although logistic regression showed good overall accuracy (90.6%), our classifier showed an accuracy of 95.2%. In all cases, the convolutional neural network (CNN) classifier outperformed both alternatives, with gains in specificity and overall accuracy being most pronounced relative to threshold-based detection. These results demonstrate that the residual convolutional architecture captures event-related features that are inaccessible to linear decision boundaries.

**Figure 3:**
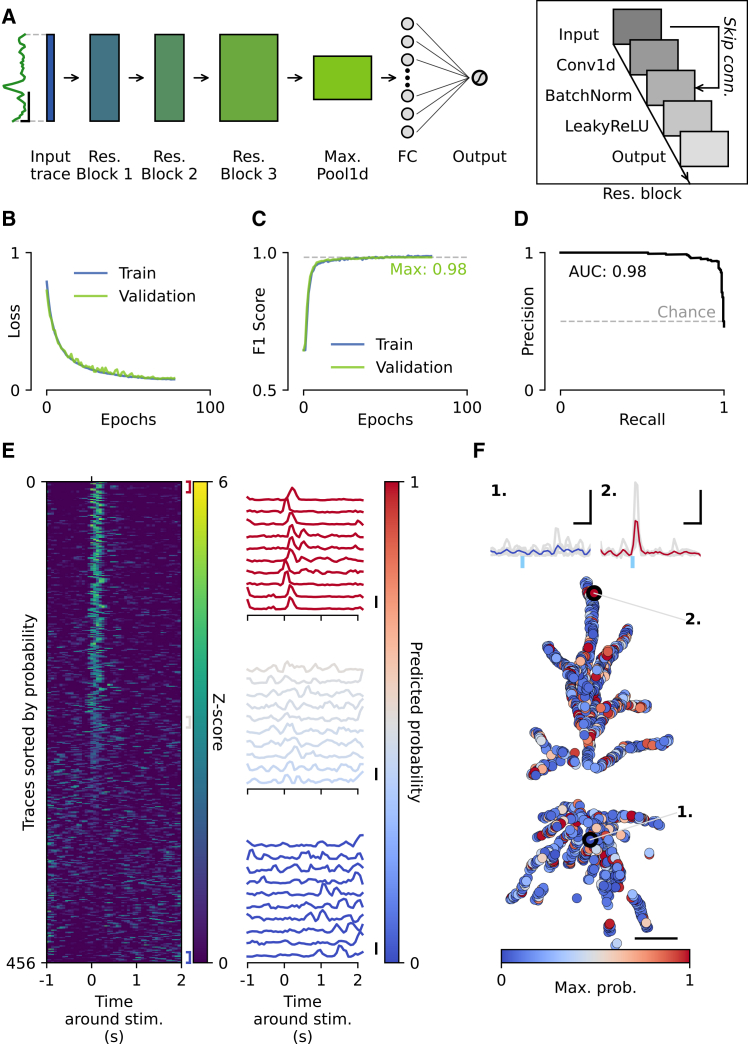
Calcium event classifier architecture and performance (A) CNN architecture. An input trace (50 time points; scale bars: 1 s, 0.1 Δ*F*/*F*_0_) is passed through three sequential residual blocks (Res. Block), followed by a 1D max-pooling layer and a fully connected (FC) layer. The output passes through a sigmoid activation, yielding a probability score. Right: each residual block includes a 1D convolutional layer, a batch normalization layer, and a leaky ReLU activation. Skip connections are added at the block output. (B) Binary cross-entropy loss for training and validation sets decreases and converges within approximately 80 epochs, with no sign of overfitting. (C) Training and validation F1 scores increase rapidly and stabilize, reaching a maximum validation F1 of 0.98. (D) Precision-recall curve on the held-out test set (AUC = 0.98). Gray dashed line indicates the performance of a random classifier for comparison. (E) Classifier output on the held-out test set. Left: heatmap of all test traces sorted by predicted event probability (top: high probability; bottom: low probability). Brackets indicate the ranges from which representative traces are drawn. Right: example traces from high (red), intermediate (gray), and low (blue) predicted probability ranges, illustrating the correspondence between the classifier score and trace morphology. Scale bars: 5 *Z* score units. (F) Spatial mapping of classifier output onto dendritic morphology. Bottom: spine centroid map from a representative neuron, color-coded by the maximum predicted event probability across 5 trials. Scale bar: 50 μm. Top: example traces from two indicated spines—spine 1 (low probability) and spine 2 (high probability). Gray, individual trials; colored lines, trial mean. Scale bars: 500 ms, 2 Δ*F*/*F*_0_.

## Discussion

Understanding how the structural organization of synaptic inputs—that is, the relative spatial positioning of co-active synapses during specific behavioral contexts—shapes dendritic integration is essential to define the rules of neuronal computations.^11^^,^^13^^,^^45^ Calcium transients within spine heads reliably report synaptic activity, yet capturing these events across the full dendritic arbor remains technically challenging. Conventional two-photon microscopes generate high-resolution imaging of spines in short and strictly in-focus dendritic segments via targeted scanning.^26^^,^^27^ To access extended dendritic structures, several 3D volumetric approaches have been developed. Electrically tunable lenses provide voltage-regulated rapid focal plane switching, allowing sequential sampling of multiple depths at sub-hertz to low-hertz volumetric rates.^46^ Beyond sequential scanning, acousto-optic deflectors (AODs) steer the focal spot to arbitrary coordinates in 3D without mechanical scanners. This allows random-access sampling of sparse neuronal populations^47^ and to record back-propagating action potentials and d-spikes from the dendritic segments of CA1 PNs at kilohertz temporal resolution^48^^,^^49^ or monitor synaptic activity in the neocortex.^50^ 3D DRIFT AO scanning extends this capability by enabling uninterrupted scanning along arbitrary 3D lines including dendritic segments.^51^ Micromirror-based systems such as scanned line angular projection^52^ (SLAP) and SLAP2^53^ sweeps the laser beam through digital micromirror devices (DMDs) to generate patterned illumination. Although these approaches enable high-speed volumetric imaging, they typically rely on sophisticated, costly, and often custom-built hardware, require specialized maintenance, and are frequently shared resources across multiple users. In contrast, conventional two-photon systems remain widely accessible but rely on sequential plane-by-plane acquisition, which is laborious, slow, and user biased, limiting the ability to assess global spatial patterns. Here, we present ROIpy and Spyne, which are Python-based pre- and post-acquisition tools designed to bridge this gap without requiring custom hardware modifications. Rather than replacing advanced volumetric systems, this pipeline transforms standard sequential layer-wise two-photon imaging into a scalable and reproducible approach for functional synaptic mapping at single-spine resolution across large dendritic domains. ROIpy utilizes dendritic reconstruction (encoded in the common SWC format) obtained by any preferred morphological tracing software to automatically tile 2D ROIs aligned with dendritic segments at the appropriate focal depth. ROI generation occurs in sub-second time, and the generated ROI files are smoothly integrated with ScanImage’s mROI acquisition tool. Structured multi-ROI post-acquisition analysis is handled by Spyne, which integrates multiple segmentation backends—DeepD3, nnU-Net, and RESPAN—providing flexibility across different imaging conditions and analysis needs. Existing tools for spine identification often require high-resolution 3D images for fine morphological study^54^ or rely on proprietary environments (e.g., Matlab).^55^ In contrast, Spyne’s modular architecture provides flexibility and compatibility within a Python-based pipeline. Through automated segmentation, we located up to thousands of spines and determined their position across branches. Detecting stimulus-evoked calcium events in this data structure (short t-series) poses a distinct classification challenge. Classical approaches such as thresholding, peak detection, deconvolution, or linear classifiers are generally optimized for continuous recordings and are sensitive to baseline shifts and variable noise. Given the structure of our data—with thousands of short, discrete, and normalized t-series with variable signal-to-noise ratios—we trained a custom CNN to detect stimulus-evoked events. The convolutional classifier provides a more robust solution, achieving performance comparable to or exceeding human annotators (Figure S10E). Several extensions of the current framework are worth highlighting. We showcased our approach using acute slice patch-clamp recordings. Although we demonstrate the approach in hippocampal CA1 PNs, ROIpy and Spyne are not restricted to this preparation. The method is applicable to neocortical circuits, where two-photon imaging of dendritic spines is well established.^56^^,^^57^ ROIpy and Spyne are also well suited for all-optical approaches. Sparse expression strategies, e.g., co-infection with a Cre-dependent GCaMP and a low-titer Cre-expressing virus, enable genetically selective labeling of neurons and help resolve single-neuron morphology in dense neuropil. Future implementations may benefit from vectors co-expressing GECIs and membrane-tethered fluorophores (separated by linkers) under the control of a common promoter to simplify cell identification and morphology tracing. To target multiple afferent populations independently, spectrally distinct opsins—for example, a blue-light-sensitive channel rhodopsin paired with a red-shifted variant—can be expressed in separate inputs, enabling pathway-selective stimulation converging onto the same neuron, with appropriate dichroic mirror configurations to separate excitation wavelengths. Finally, the pipeline can be adapted to guide sequential targeted scanning in 2D multiphoton setups *in vivo*. This could support functional synaptic mapping based on behavioral tasks or sensorial stimulation. The final output of the workflow is a functional input map with single-spine resolution. This positions our method as complementary to the structural techniques such as fluorescence imaging methods^58^ or electron microscopy,^59^ which yield dense maps but lack functional readouts. Similarly, GRASP^21^^,^^22^^,^^60^ relies on interaction with specific presynaptic partners but does not capture activity. Critically, none of these approaches can report dynamic, activity-dependent processes under physiological or experimental conditions. Our pipeline identifies individual spines activated by the defined stimulation paradigms and, because measurements can be repeated across sessions, is well suited for plasticity studies—addressing questions about how the spatial organization of synaptic inputs is established, maintained, and modified over time.

### Limitations of the study

Several limitations of the present approach stem from its reliance on sequential dendritic sampling, which requires extended acquisition times when reconstructing large arbors. In the context of patch-clamp recordings, this is particularly consequential. While a patch-clamp setup offers access to somatic subthreshold dynamics and control over resting membrane potential, it entails up to 2–3 h of continuous recording for full arbor sequential scanning, straining seal stability and increasing the experimental failure rate. Mechanical instability is negligible over the short stimulation window (3.125 s per z layer), as perfusion or pipette-related movement is minimal on this timescale. However, slow drifts accumulating during such long experiment can cause misalignment between the acquired and original reference images, leading to out-of-focus ROIs and, ultimately, gaps in the spine map. The completeness of the resulting map is further constrained by the quality of the initial dendritic reconstruction. ROI placement depends on the labeling efficiency and imaging signal-to-noise ratio, which vary with preparation quality. When neurons are loaded via patch pipette, incomplete filling of distal or fine-caliber branches may result in partial arbor sampling. The temporal structure of the acquisition—consisting of short, stimulus-locked recordings—is a deliberate design choice with its own trade-offs. While such traces are optimized for detecting evoked events, they do not capture continuous activity dynamics. Although this is well suited for the mapping paradigm presented here, the approach is not directly applicable to studies of spontaneous activity or sustained network states without modification to the acquisition and analysis framework. Adaptation to *in vivo* recordings would require additional processing steps not implemented in the current framework, such as motion correction and back-propagating action potential subtraction to unmix the spine calcium signals from global dendritic activity.^14^ These steps were omitted here given the mechanical stability of acute slice preparations and low probability for neurons to spontaneously fire, but they represent a prerequisite for reliable *in vivo* deployment. Combining optogenetic stimulation with multiphoton calcium imaging requires protection of the photon detectors from the stimulation light. We used gated PMTs, which transiently disable signal collection during light-emitting diode (LED) illumination. To compensate for gating-induced image loss, we applied a gating artifact correction that identifies and interpolates the affected scan lines. The information loss was modest: the gating delay was set to 6 ms following LED onset—within the range of a synaptic delay—preserving event onset detection. In cases where the peak of a calcium transient falls partially within the gating window, the slow decay kinetics of Fluo-5F^61^ allow reliable detection in subsequent frames, ensuring accurate event reconstruction. Nonetheless, in cases where calcium transients overlap substantially with the gating window, partial signal loss cannot be fully excluded. The spatial resolution of two-photon imaging also imposes inherent limits on spine identification. Comparison with stimulated emission depletion (STED) microscopy has shown that approximately 23% of spines resolved by two-photon imaging correspond to two distinct spines at super-resolution,^62^ meaning that some detected events may reflect the compound activity of closely apposed inputs, rather than a single synapse. This should be considered when interpreting maps of apparent co-activation. Because acquisition is performed sequentially at discrete *z* planes, the spatial representation of spine positions in the resulting maps carries an additional geometric limitation. While the *xy* position of each detected spine is determined by segmentation, the *z* coordinate is assigned the ROI’s discrete imaging plane z depth. However, this does not prevent branch-wise spine assignment, i.e., mapping spines to their parent branch. Furthermore, spines are imaged only along a single 2D *z* plane; therefore, no single-spine 3D volumetric reconstruction is performed. Taken together, these limitations are largely tractable. Drift correction, motion compensation, and continuous activity monitoring are addressable through extensions to the current framework, and their resolution would substantially broaden the range of experimental contexts to which the pipeline applies.

## Resource availability

### Lead contact

Requests for further information and resources should be directed to and will be fulfilled by the lead contact, Dr. Andrea Barberis (andrea.barberis@iit.it).

### Materials availability

This study did not generate new unique reagents.

### Data and code availability

• Data: The following data have been deposited at IIT-Dataverse and are publicly available as of the date of publication at https://doi.org/10.48557/GZYED8:
  ○ Training images and spine and dendrites labels used to train spine segmentation algorithm.
  ○ Trained DeepD3 and nnU-net model.
  ○ High- and low-signal-to-noise ratio images used to train CSBDeep model.
  ○ Trained CSBDeep model.
  ○ Calcium imaging traces used to train custom deep learning classifier.
  ○ Calcium event classifier trained model.
• Code: All original code has been deposited on GitHub at https://github.com/dcupolillo/ROIpy (https://doi.org/10.5281/zenodo.21334250), https://github.com/dcupolillo/spyne (https://doi.org/10.5281/zenodo.21334656), and https://github.com/dcupolillo/calcium_event_classifier (https://doi.org/10.5281/zenodo.21334890).
• Additional information: Any additional information required to reanalyze the data reported in this paper is available from the lead contact upon request.

## Acknowledgments

We thank Tommaso Fellin for critically reading the manuscript. We thank Mattia Pesce for technical help. We thank Maithé Loisy, Niccolò Mattiello, Belén Hervas Povo, and Cecilia Rostagny for manual annotations. The author(s) declare that financial support was received for the research, authorship, and/or publication of this article. The present work has been supported by HORIZON-MSCA-2021-PF-01 –SynEMO (project: 101068871).

## Author contributions

Conceptualization, D.C., V.R., and A.B.; methodology, D.C., V.R., and A.B.; investigation, D.C., V.R., and V.G.; software, D.C.; data curation, D.C.; writing – original draft, D.C. and V.R.; writing – review & editing, D.C., V.R., and A.B.; funding acquisition, D.C. and A.B.; resources, D.C. and A.B.; supervision, A.B.

## Declaration of interests

The authors declare no competing interests.

## Declaration of generative AI and AI-assisted technologies in the writing process

During the preparation of this work, the author(s) used GPT-5.2 and Claude Sonnet 4.6 to improve readability. After using this tool or service, the author(s) reviewed and edited the content as needed and take(s) full responsibility for the content of the publication.

## STAR★Methods

### Key resources table

**Table:** 

REAGENT or RESOURCE	SOURCE	IDENTIFIER
**Bacterial and virus strains**		

AAV5-CaMKIIa-hChR2(H134R)-EYFP	Addgene viral prep # 26969-AAV5	RRID: Addgene_26969

**Chemicals, peptides, and recombinant proteins**		

N-Methyl-D-glucamine	Sigma-Aldrich	Cat#66930; CAS: 6284-40-8
Potassium chloride	Sigma-Aldrich	Cat#P9333; CAS: 7447-40-7
Sodium phosphate monobasic monohydrate	Sigma-Aldrich	Cat#S9638; CAS: 10049-21-5
Sodium bicarbonate	Sigma-Aldrich	Cat#S5761; CAS: 144-55-8
HEPES	Sigma-Aldrich	Cat#H3375; CAS: 7365-45-9
Glucose	Sigma-Aldrich	Cat#G7021; CAS: 50-99-7
Thiourea	Sigma-Aldrich	Cat#T7875; CAS:62-56-6
Sodium ascorbate	Sigma-Aldrich	Cat#A7631; CAS:134-03-2
Sodium pyruvate	Sigma-Aldrich	Cat#P2256
Calcium chloride dihydrate	Sigma-Aldrich	Cat#C5080; CAS: 10035-04-8
Magnesium sulfonate heptahydrate	Sigma-Aldrich	Cat#M2773; CAS: 10034-99-8
Sodium chloride	Sigma-Aldrich	Cat#S9888; CAS: 7647-14-5
Magnesium chloride solution	Fluka	Cat#63020; CAS: 7786-30-3
Calcium chloride solution	Fluka	Cat#21114; CAS: 10043-52-4
Picrotoxin	Sigma-Aldrich	Cat#P1675; CAS: 124-87-8
D-serine	TOCRIS	Cat#0226; CAS: 312-84-5
CNQX	Sigma-Aldrich	Cat#C239; CAS: 115066-14-3
Potassium D-gluconate	Sigma-Aldrich	Cat#G4500; CAS: 299-27-4
Phosphocreatine disodium salt hydrate	Sigma-Aldrich	Cat#P7936; CAS: 19333-65-4
EGTA	Sigma-Aldrich	Cat#E3889; CAS: 67-42-5
ATP magnesium salt	Sigma-Aldrich	Cat#A9187; CAS: 74804-12-9
GTP sodium salt hydrate	Sigma-Aldrich	Cat#G8877; CAS: 36051-31-7
Alexa Fluor 594 Hydrazide	Thermofisher	Cat#A10438

**Deposited data**		

Allen Cell Types Database^38^	Allen Institute	RRID: SCR_014806
Database: Models and training data	This paper	https://doi.org/10.48557/GZYED8

**Experimental models: Organisms/strains**		

Mouse: Fos^2A-iCreERT2^	The Jackson Laboratory	RRID: IMSR_JAX:030323

**Software and algorithms**		

DeepD3	Fernholz et al.^31^	deepd3.forschung.fau.de
RESPAN	Bernal-Garcia et al.^33^	github.com/lahammond/RESPAN
CSBDeep	Weigert et al.^42^	github.com/CSBDeep/CSBDeep
nnU-net	Isensee et al.^32^	RRID:SCR_028165
Scanimage 2021	MBF Bioscience; Pologruto et al.^30^	RRID:SCR_014307
pCLAMP 11	Molecular Devices	RRID:SCR_011323
Fiji	Schindelin et al.^35^	RRID:SCR_002285
Single Neurite Tracer plugin	Arshadi et al.^34^	github.com/morphonets/SNT
Python 3	python.org	RRID:SCR_008394
Allen Institute Software Development Kit	Allen Institute	github.com/AllenInstitute/AllenSDK
scikit-learn	scikit-learn.org	RRID:SCR_002577
pytorch	pytorch.org	RRID:SCR_018536
pyABF	swharden.com/pyabf	github.com/swharden/pyABF
miniML	O’Neill et al.	github.com/delvendahl/miniML
tifffile	pypi.org/project/tifffile	RRID:SCR_023338
numpy	numpy.org	RRID:SCR_008633
scipy	scipy.org	RRID:SCR_008058
scikit-posthoc	github.com/maximtrp/scikit-posthocs	RRID:SCR_021363
matplotlib	matplotlib.org	RRID:SCR_008624
ROIpy	This paper	github.com/dcupolillo/ROIpy https://doi.org/10.5281/zenodo.21334250
Spyne	This paper	github.com/dcupolillo/Spyne https://doi.org/10.5281/zenodo.21334656
Calcium event classifier	This paper	github.com/dcupolillo/calcium_event_classifier https://doi.org/10.5281/zenodo.21334890

### Experimental model and study participant details

#### Animals

All animal care and experimental procedures were conducted in accordance with Istituto Italiano di Tecnologia (IIT) licensing as well as the Italian Ministry of Health (D.Lgs 26/2014) and EU guidelines (Directive 2010/63/EU). The experiments were conducted on Fos-TRAP2^63^ (Fostm2.1(iCRE/ERT2) Luo/J, Jackson laboratory, USA) transgenic mice of either sex aged 5 to 7 weeks. Male and female inbred Fos-TRAP2 mice were housed in filtered cages, in a climate-controlled animal facility (22 ± 2°C) and maintained on a 12-h light/dark cycle with water and food *ad libitum*.

### Method details

#### Virus injection

Mice were first weighed and checked for overall health status. Subsequently, they were placed for 3 min in an anesthesia chamber with isoflurane (4%) and 95% oxygen (1.5 L/min). Mice were then mounted on a stereotaxic frame under 2% isoflurane (0.8L/min). Injection of ketorolac (5–7.5 mg/kg) and enrofloxacin (5 mg/kg**)** were provided intraperitoneally as analgesic and antibiotic, respectively. During the surgery, the temperature was maintained at 37°C using a feedback-controlled heating pad (RWD Life Science Co., Ltd) and ophthalmic ointment was applied to avoid eye drying. A small craniotomy was performed with a dental drill (RWD Life Science Co., Ltd). AAV-CaMKII-hChR2-EYFP (titer >10^13^ vg/ul, Addgene: # 26969-AAV5) was injected bilaterally in basolateral amygdala (stereotactic coordinates relative to bregma: AP +1.6, ML: ±3.32, DV: +4.90) through a glass pipette attached to a microsyringe (1701RN, Hamilton Company, Reno, NV, USA) at a rate of 60 nL/min controlled with a microinjection syringe pump (UMP3, World Precision Instruments, Sarasota, FL, USA). Following infusion, the injection syringe was left in place for 10 additional minutes before withdrawal. Mice were then monitored for two days post-surgery to ensure proper recovery. Mice were allowed to recover for 4 to 6 weeks.

#### Acute slice preparation

Mice were deeply anesthetized by injecting intraperitoneally ketamine (200 mg/kg) and xylazine (20 mg/kg) and transcardially perfused with an ice-cold N-methyl-D-glucamine (NMDG)-based solution^64^ containing (in mM): 92 NMDG, 2.5 KCl, 1.25 NaH_2_PO_4_, 30 NaHCO_3_, 20 HEPES, 25 glucose, 2 thiourea, 5 Na-ascorbate, 3 Na-pyruvate, 0.5 CaCl_2_·2H_2_O, and 10 MgSO_4_·7H_2_O, 300 mOsm/Kg, pH adjusted to 7.3–7.4 with 5M hydrochloric acid and equilibrated with 95% O_2_ and 5% CO_2_. Brains were extracted and submerged in ice-cold NMDG-cutting solution. Horizontal brain slices (350 μm thick) were prepared from either hemisphere in ice-cold NMDG-cutting solution using a vibratome (VT1200S, Leica Microsystems), incubated in a maintenance chamber at ∼35 °C for 20/30 min in the NMDG-cutting solution, and subsequently stored at room temperature (RT) in artificial cerebrospinal fluid (ACSF) until recording. The ACSF solution contained (in mM): 125 NaCl, 26 NaHCO_3_, 2.5 KCl, 1.25 NaH_2_PO_4_, 10 glucose, 2.3 CaCl_2_, and 1.3 MgCl_2_, equilibrated at 95% O_2_ and 5% CO_2_ for at least 40 min.

#### Patch-clamp electrophysiology

Patch clamp experiments were performed similarly to previous reports.^57^ After at least 1h of recovery in ACSF, slices were transferred to a recording chamber and constantly perfused with a zero-Mg^2+^ ACSF (2 mL/min) at RT, to allow for the removal of the Mg^2+^ block of NMDA receptors. Picrotoxin (50 μM), d-serine (10 μM), and CNQX (10 μM) were present in the superfusate of all experiments to ensure that Ca^2+^ events originated from monosynaptic glutamate transmission. Patch pipettes were prepared using a horizontal puller (P-1000 Next Generation Micropipette Puller, Sutter Instrument) from borosilicate glass capillaries (Warner Instruments, LLC, Hamden, USA). When filled with a K-gluconate-based intracellular solution, pipette resistance was 4–6 MΩ. The composition of the intracellular solution was as follows (in mM): 135 K-gluconate, 5 KCl, 2NaCl, 10 Na2P-Cratine, 0.1 EGTA, 10 HEPES, 5 Mg-ATP, 0.4 Na-GTP (285 mOsm/Kg, adjusted to pH 7.2 with KOH). In addition, Alexa Fluor 594 (75 μM, ThermoFisher #A10438) and the synthetic calcium indicator Fluo-5F pentapotassium salt (300 μM, ThermoFisher #F14221) were added to the intracellular solution. Cells were identified by a Dodt Gradient Contrast (DGC) microscope (Hyperscope, Scientifica, UK) in bright-field using a UV filter paired with an oil-condenser (Olympus U-AAc), using a 16× water-immersion objective lens (Nikon). Whole-cell configuration was reached in identified and selected pyramidal neurons in ventral CA1. Holding current and access resistance were continuously monitored throughout the experiment. Cells were discarded if holding current was >200 pA and if access resistance >20 MΩ or if these parameters changed by more than 25% during recording. No liquid-junction potential correction was used. Currents were sampled at 10 KHz using the 700B Axopatch amplifier (Molecular Devices, Sunnyvale, CA) and low-pass filtered at 1 kHz. For SCs stimulation, an Ag/AgCl electrode was introduced in an ACSF-filled glass pipette and positioned approximately 300 μm from the patched cell in the stratum radiatum. To elicit a synaptic response, we delivered squared paired pulses at 20 Hz, consisting of two stimuli (0.8 ms duration, separated by 50 ms) to minimize synaptic failures probability. The intensity of stimulation was adjusted by using an isolated constant-current stimulator (Digitimer Ltd.) to elicit a minimal synaptic response. For optogenetics, single 2 ms-long LED pulse at 470 nm (CoolLED’s pE-300^white^) with an intensity of 3–7 mW (measured below the objective lens) were delivered and band-passed through a GFP filter (SCI-49002s, Scientifica, UK).

#### Two-photon scanning system

The optic path consists of an excitation source provided by a femtosecond Ti:Sapphire pulsed laser (Chamaleon Ultra II, Coherent Inc.), a modulatory Pockel’s cells (Conoptics, Dambury CT), beam expander, mechanical shutter, and HyperScope (Scientifica UK) Scan Path containing tube lens, beam combiner. The two-photon microscope (SliceScope, Scientifica UK) was equipped with a dual-axis galvo-galvo module (HyperScope Y-galvanometer coupled with an additional HyperScope Resonant X-Module) and GaAsP photomultipliers (Hamamatsu). The laser was tuned to 830 nm to simultaneously excite A594 and Fluo-5F, with a power of 6–11 mW measured under the objective. This power was kept constant throughout the imaging depth. Simultaneous two-photon excitation and the LED optogenetic stimulation were enabled by a custom primary dichroic mirror (ZT590-180dcrb, Chroma Technology Corp.). Emission signals were separated using a 580 nm dichroic mirror and detected through bandpass filters centered at 525/50 nm (Fluo-5F) and 620/60 nm (A594). Frames were acquired through a water-immersion objective lens (Nikon 16×/0.8 NA/3 mm WD) using ScanImage software (MBF Bioscience, Williston, VT). The signal was digitized in pixels with intensity values as signed 16-bit integers (−32,768 to 32,767).

#### Reference volume acquisition and morphological reconstruction

After obtaining a stable whole-cell configuration, cells were allowed to load with A594-containing intracellular solution for at least 20 min to ensure adequate filling of dendritic processes, including thin and distal branches. The depth range containing the whole dendritic arborization was measured and, using the ScanImage Stack Acquisition tool, a bounded z stack was configured. The number of linearly spaced slices was adjusted to yield a z-step of 1–1.5 μm, balancing the trade-off between axial resolution and total number of slices. Frames (1024 × 1024 pixels) were acquired at 0.27 Hz without averaging. The stack image was then used as reference to semi automatically trace the neuronal morphology using SNT. A single point soma was created, and then the traced dendrites were manually tagged as apical or basal.

#### ROI design and optimization

ROIpy parses SWC-written 3D neuronal morphologies into graph representations of interconnected nodes (with associated metadata). Coplanar nodes were grouped based on continuous connectivity, forming scattered chains which served as the basis for automated placement of rectangular ROIs. For each node chain, the straight line connecting the nodes closest to and farthest from the soma defined the longitudinal axis of the rectangular ROIs, determining its height and rotation. Rectangles were symmetrically expanded to a fixed width of 10 μm perpendicular this axis, ensuring the dendrite remained centered along it. ROIs were then refined as described previously. All ROIs processing parameters are tunable and are summarized in Table S1 ROIs with extreme aspect ratios (width-to-height >3.5) were excluded unless they were contiguous with other ROIs. ROIs were symmetrically elongated by 33% of their height. To identify spatially overlapping ROIs, we created a pairwise overlap matrix for every z-layer. ROIs with more than 90% of their area covered by another ROI were discarded. A dual-radius exclusion zone was applied to filter out dendritic ROIs located within 20 μm (basal) or 50 μm (apical) around the soma. To allocate the appropriate number of pixels per ROI, a target frame rate value was assigned (e.g., 16 Hz), and the corresponding scan period was computed as the inverse of the frame rate. We calculated the scan time per frame based on acquisition parameters—i.e. sampling rate, pixel dwell time, bin factor, fly-to and fly-back duration, and fill fraction (Equation 1). For each layer, we calculated the active scanning time (*T*_*active*_) by subtracting the fly-to periods between ROIs and the frame-flyback to scan start position period, both initially set at 1 ms. The total active scan area (*A*_*scan*_) was calculated by summing the area of all ROI, corrected by the fill fraction (0.9) to account for overscan required for bidirectional scanning. Using a predefined pixel dwell time (*T*_*dwell*_) of 3.2 μs, we computed an initial pixel size (*S*_*pixel*_) as:

(Equation 1) $$S_{pixel}=\sqrt{\frac{A_{scan}\times T_{dwell}}{T_{active}}}$$

The frame rate that resulted from such pixel dimension was calculated and compared to the target frame rate. Pixel size was iteratively refined in a convergence loop (up to 100 iterations), to achieve the target frame rate (Equation 2) with a tolerance of 0.3 Hz. To calculate frame rate (*R*_*frame*_), we multiplied the allocated number of pixels by *T*_*dwell*_ for each ROI, added non-active scan times:

(Equation 2) $$R_{frame}=\frac{1}{\sum_{i=0}^{ROI_{n}}T_{ROI_{i}}+\left[T_{fly-to}\times \left(n_{ROI}-1\right)\right]+T_{flyback}}$$

and finally ensured that the computed pixel dimensions remained compatible with both the digitizer’s acquisition rate and the two-photon sampling rate. This correction provided the actual scan duration per line, which was used to validate that the total scan time aligned with the desired frame period. Pixel dimensions were prevented from exceeding the diffraction-limited resolution of the optical instrument by defining an upper limit according to Abbe-Rayleigh’s criteria^65^:

(Equation 3) $$d=1.22\times \frac{\lambda }{2NA}=0.61\times \frac{\lambda }{NA}$$

The minimum resolvable distance (*d*) between two objects depends on the numerical aperture *NA* of the microscope objective and the imaging wavelength *λ*. In line with Nyquist sampling criteria, the pixel size (*f*_*s*_) needs to be 2–3 times smaller than the microscope resolution (*f*_*MAX*_):

(Equation 4) $$f_{s}=\frac{1}{2\times f_{\mathrm{MAX}}}$$

therefore, if pixel-to-μm ratio exceed this constraint during pixel dimension adjustments, ROIs are split into two or more separate scanning groups (and consequently *roi* files) located within the same z-layer, thus reducing the total scanning area per group. Fine frame rate adjustments were made by modifying flyback duration, that is, the time required for the laser to return to the starting position after each frame.

#### Allen cell-type dataset

We queried the Allen Cell-Type Database (https://celltypes.brain-map.org) using an application programming interface (API) written in Python, using the *allensdk* library, to retrieve mouse neurons with available morphological reconstructions. The search was restricted to layer 5 neurons in the primary visual area (VISp). A total of 63 neurons were retrieved, and their corresponding SWC files were used for ROIpy-based processing and reconstruction. Table S2 summarizes the retrieved neurons.

#### Computational resources

All deep learning models were trained and evaluated on a workstation equipped with an NVIDIA GeForce RTX 3080 GPU (10 GB VRAM) and 128 GB RAM. Model training and inference were performed using both *PyTorch* and *TensorFlow*, depending on package requirements.

#### DeepD3 training and inference

We trained the segmentation model using a dataset of two-dimensional ROIs using the provided DeepD3 suite for training own data. The dataset consisted of 27 images of dendrites and spines characterized by an average pixel-to-μm ratio of 0.035 ± 0.010 μm, aligning with the range of the collected data (Figure S4B). Images were manually annotated with pixel precision using *Napari* to generate binary masks of spines and dendrites (Figure S4A). To promote detection of perpendicularly protruding spines, we labeled bright, bulbous features along the dendritic shaft as spines. 2D images and corresponding annotations were adapted to DeepD3 data streamer by introducing them in a dummy 3D stack (x, y, 1). Image stacks were preprocessed by subtracting the minimal pixel value from each frame and converted to unsigned 16-bit integers. Data was split into training and validation sets (0.8/0.2). Training was performed in line with DeepD3 provided toolbox. Training data were generated by extracting 128 × 128-pixel patches (*n* = 10000). An adaptive content threshold was determined empirically by analyzing label density across patches and selecting the 10^th^ percentile value. The training generator employed data augmentation and random shuffling. The model was trained with the following hyperparameters: batch size of 32, base filter count of 32, initial learning rate of 5 × 10^−4^, and a total of 30 epochs. Model was evaluated using a combined Dice and Mean Squared Error loss (*tf.keras.losses.dice_loss* and *tf.keras.losses.MeanSquaredError*) while monitoring accuracy and Intersection-over-Union (IoU). To optimize convergence, we employed an exponential learning rate decay (*keras.callbacks.LearningRateScheduler*) after the 15^th^ epoch. The training hyperparameters are summarized in Table S3. For bulk inference, images were padded to a common size, defining height and width as the smallest multiple of 32 pixels that exceeded the largest image dimensions in the dataset. The spine and dendrites probability maps resulting from inference were then post-processed to generate final segmentations masks. Maps were binarized using a threshold of 0.3 and 0.7 for spines and dendrites respectively and spine labels smaller than 3 pixels were discarded. To eliminate segmentation artifacts disconnected from dendrites, a 12-fold dilation of the dendritic mask was applied (*scipy.ndimage.binary_dilation*) and spine labels falling outside this boundary were removed. Additionally, to prevent artificial merging of spine masks at diagonal junctions, 2 × 2 windows with diagonally opposed non-zero pixels were set as 0. To separate closely apposed spines, a distance transform (*cv2.distanceTransform*) followed by a marker-based watershed algorithm (*skimage.segmentation.watershed*) using local maxima (*skimage.feature.peak_local_max*) was applied. The resulting masks were labeled (*skimage.measure.label*), and pixel area and centroid coordinates were calculated for each spine. Centroids were projected to FOV reference space coordinates using affine transformations.

#### CSBDeep model training for CARE

We trained a CSBDeep model for content-aware image restoration using artificial degradation of a high-quality ground truth dataset of dendritic images. To simulate low-light imaging conditions characteristic of fluorescence microscopy, wefirst scaled down by a factor of 10 to simulate reduced photon counts. Optical blur from the microscope’s point spread function (PSF) was modeled using a 2D Gaussian kernel. PSF parameters were calculated based on the diffraction limit using Abbe’s formula (Equation 3). The FWHM was converted to the standard deviation of a Gaussian function and scaled to pixel units for each image. The PSF kernel was applied via FFT-based convolution. Photon shot noise was simulated by applying Poisson noise. Detector readout noise and other electronic noise sources were modeled by adding Gaussian noise with a standard deviation of 50. Training data was generated by extracting 128 × 128 pixel patches from paired low-quality and ground truth images. Patches containing minimal foreground content (less than 40% above background threshold) were excluded. A total of 32 patches were extracted per image pair and split into training (75%) and validation (25%) sets. Data augmentation was performed during training to improve model generalization, consisting of random horizontal flips, vertical flips, and 90° rotations applied with 50% probability to each training batch. We employed the CARE U-Net-based convolutional neural network architecture with a depth of 2 levels, 5 × 5 convolution kernels, and 32 initial feature channels. The model was trained for 60 epochs using the Adam optimizer with an initial learning rate of 4 × 10^−4^. The learning rate was reduced by a factor of 0.5 if validation loss did not improve for 10 consecutive epochs. Training was performed with a batch size of 16 and 400 steps per epoch. Mean squared error (MSE) served as the loss function, with mean absolute error (MAE) monitored as an additional metric. Model performance was evaluated on the validation set after each epoch to detect overfitting.

#### Manual annotations of dendritic spines

To identify the location of spines and dendrites in 2D images, we asked 5 human annotators to independently manually label dendritic spines with pixel precision from 5 images. The task was carried out using a custom *Napari* GUI. Annotators were minimally instructed to label spine regions based on morphological of the spine head. The annotated dataset spans a range of imaging conditions and spine morphologies representative of the experimental data.

#### Gated PMTs artifact correction

During optogenetic stimulation, scattering blue light used to excite ChR2 is collected by the PMTs alongside fluorescence from excited functional indicators (Fluo-5F and A594). To protect from photodamage, PMTs equipped with gating function are employed. The gate circuit temporarily disables photon detection, electrically protecting the PMTs from excessive light exposure. The gate circuit is triggered by the Digidata 3 ms before LED onset and has a duration of 10 ms—longer than the 2 ms-long light pulse—usually within the acquisition time of a single frame of a ROI (Figure S5A). Due to bidirectional scanning, the gating interval results in a horizontal stripe of low-intensity pixels acquired during the gating period (Figure S5C). Gating artifact correction is performed as a pre-processing step. In few words, for each frame in the Alexa Fluor channel, the average intensity value of every line (row) is computed (Figure S5C). Lines affected by gating are identified as those whose average intensity deviates by more than 3 standard deviations (*σ*) from the mean intensity of all lines of that frame (Figure S5C). Deviating lines are then corrected by interpolating each pixel values with the average of the corresponding pixels from preceding and subsequent frame.

#### Calcium imaging and analysis

Frames (*n* = 50), which consisted of a set of coplanar ROIs, were acquired at 16 Hz, for a total duration of 3.125 s. Frames were 3D-median filtered using a 3D kernel with spatial dimension of 0.3 μm adjusted across z planes yielding different pixel dimensions and a temporal dimension of 3 frames. Functional signals were extracted from spine masks. A raw t-series was generated by averaging intensity of all pixels included in the mask. For each ROI, fluorescence traces were corrected for local background (neuropil) contamination by subtracting a scaled background signal estimated from a surrounding region. The background signal was computed as the median fluorescence in the area outside a dilated spine and shaft mask (disk dilation = 6), and a scaled fraction (0.7) was subtracted frame-by-frame. Δ*F*/*F*_0_ was calculated as:

(Equation 5) $$\frac{\Delta F}{F_{0}}=\frac{F_{t}-F_{0}}{F_{0}}$$

where *F*_*t*_ is the background-corrected average fluorescence intensity at time *t* and *F*_0_ is the baseline trace computed as the 10^th^ percentile of *F*_*t*_ within a running kernel of 0.5 s centered in *t*. For each trace, we calculated standardized z-scores as:

(Equation 6) $$Z_{score}=\frac{F_{t}-\mu }{\sigma }$$

where *μ* is the average of all *F*_*t*_ and *σ* is the standard deviation of *F*. For data loading to the calcium event classifier during training, the *Z* score is calculated using:

(Equation 7) $$Z_{score}=\frac{F_{t}-\eta_{b}}{MAD}$$

(Equation 8) MAD=median(|Fb−ηb|)

Where *η*_*b*_ is the median of the baseline *F*_*b*_.

#### Calcium event classifier training and inference

To train a classifier to identify active synapses, we built a ground-truth dataset using 2204 Δ*F*/*F*_0_ traces randomly subsampled from recordings across various neurons. To add variability and increase model robustness, extracellular stimulation was delivered using either electrical pulses or optogenetic blue light pulses. In all cases, pulses were triggered 1 s after scan onset via hardware synchronization, generating time-locked calcium peaks. Traces were annotated as 0 or 1 by experts using a custom GUI written in Python. Annotation was performed based on both the characteristic dynamics of Ca^2+^ transients and their temporal occurrence relative to the stimulation trigger. Data was divided into training and validation sets (0.75/0.25) maintaining class ratio in both splits (*sklearn.model_selection.train_test_split*). Before loading data to the network (*torch.utils.data.DataLoader* with *SubsetRandomSampler*), traces were preprocessed. Each Δ*F*/*F*_0_ trace was baseline-normalized by subtracting the median of the baseline (frames 3–15) and divided by the median absolute deviation (*MAD*) (Equations 7 and 8). During training, data augmentation was applied to the training set with 50% probability by adding Gaussian noise (σ = 0.5 × baseline MAD) to the baseline segment The classifier was conceptualized around a residual CNN architecture with batch normalization, designed for binary classification task of one-dimensional input data. The code for training was written in Python, using the deep learning library *PyTorch*. The neural network comprises three residual blocks, each containing a convolutional layer (*torch.nn.Conv1d*), batch normalization (*torch.nn.BatchNorm1d*), and leaky rectified linear unit activation (*torch.nn.LeakyReLU*) with skip connections for improved gradient flow. After the convolutional blocks, a single max-pooling layer (*torch.nn.MaxPool1d*) with dropout provide spatial downsampling and regularization. The flattened features are fed to a single fully connected layer (*torch.nn.Linear*) that outputs a logit. The network was trained using a binary cross-entropy loss function (*torch.nn.BCEWithLogitsLoss*) between predicted logits and labels. During training, this logit is passed through a sigmoid activation function (*torch.sigmoid*) to yield probability scores. Training was performed with early stopping to terminate training when the validation loss failed to improve for 10 consecutive epochs. In addition, we implemented a learning rate scheduler (*torch.optim.lr_scheduler.ReduceLROnPlateau*) that reduced the learning rate by a factor of 0.5 when validation loss plateaued for 5 epochs. The specific hyperparameters are summarized in Table S5. During training, model performance was monitored at each epoch using the F1 score:

(Equation 9) $$F1_{score}=2\times \frac{precision\times recall}{precision+recall}$$

Where precision and recall are:

(Equation 10) $$precision=\frac{TP}{TP+FP}$$

(Equation 11) $$recall=\frac{TP}{TP+FN}$$

Here, *TP*(true positives) and *TN* (true negatives) are correct predictions, while *FP* (false positives) and *FN*(false negatives) represent misclassifications. The test dataset consisted of 456 held out Δ*F*/*F*_0_ traces. In all cases, the decision boundary for predicted probability values was selected by computing the F1 score across a range of thresholds and selecting the value that maximized it.

#### Threshold-crossing calcium event classification

For each z-scored t-series, we computed the standard deviation (*σ*) and a detection threshold of either 1.96 × *σ* (containing 95% of data point of a standard normal distribution) or a more conservative 2.5 × *σ* (containing >98% of data point of a standard normal distribution) above mean baseline value. Standard deviation was calculated in a time window of 0.625 s prior to the stimulation onset. A time window of 0.375 s following the stimulation onset was defined as the detection range. Traces were baseline-zscored (Equation 7) and classified as containing a calcium event if the signal exceeded the threshold for at least 2 consecutive time points within this window.

#### Logistic regression classifier

The same labeled dataset used to train the deep neural network was used to train a logistic regression classifier (*sklearn.linear_model.LogisticRegression*) that expresses probability of containing a calcium event as:

(Equation 12) $$P\left(y=1|x\right)=\frac{1}{1+e^{-\left(wX+b\right)}}$$

where *X* is the Δ*F*/*F*_0_ time series (z-scored as in Equation 7), *w* is a weight vector, and *b* is the intercept. Data were split and stratified into training (80%) and validation (20%) sets (*sklearn.model_selection.train_test_split*). Hyperparameters, including the inverse regularization strength C and the L2 penalty, were optimized via grid search (*sklearn.model_selection.GridSearchCV*) with 5-fold cross-validation. Class weights were set to ‘balanced’ to ensure equal contribution from both classes during optimization.

### Quantification and statistical analysis

Data are indicated throughout the figure legends with sample number (*n*) and mean ± standard error of the mean (SEM). Data are shown as individual points (gray circles) next to boxplots. Black crosses overlayed to data indicate the mean and SEM. Boxes represent the interquartile range (IQR) with the median marked by a horizontal line. Whiskers extend to data within 1.5 × IQR; points beyond this range are considered outliers. Statistical significance was defined as *p* < 0.05 throughout. Before any between-group comparison, the Shapiro-Wilk test was applied to each group to determine whether a parametric or non-parametric test was appropriate. For paired two-group comparisons, the paired *t* test was used when normality was met; otherwise, the Wilcoxon signed-rank test was applied. For multiple group comparisons, one-way ANOVA followed by Tukey’s HSD post-hoc test was applied when normality was met; otherwise, Kruskal-Wallis test followed by Dunn’s post-hoc test corrected for multiple comparisons using the Bonferroni method was applied. Pearson’s correlation was used to measure linear association. All statistical analyses were performed in Python using *scipy* and *scikit-posthocs*.
