# Supplementary material for: Deep learning-assisted mapping of dendritic spines using sequential 2D two-photon calcium imaging
Source: iScience. 2026 Aug 1;29(8):117010. doi: 10.1016/j.isci.2026.117010 (PMC13453581; doi:10.1016/j.isci.2026.117010)
Supplement: Supplementary file 1 [file mmc1.pdf]

iScience, Volume *29*

Supplemental information

Deep learning-assisted mapping of dendritic spines using sequential 2D two-photon calcium imaging

Dario Cupolillo, Vincenzo Regio, Valentina Galleano, and Andrea Barberis

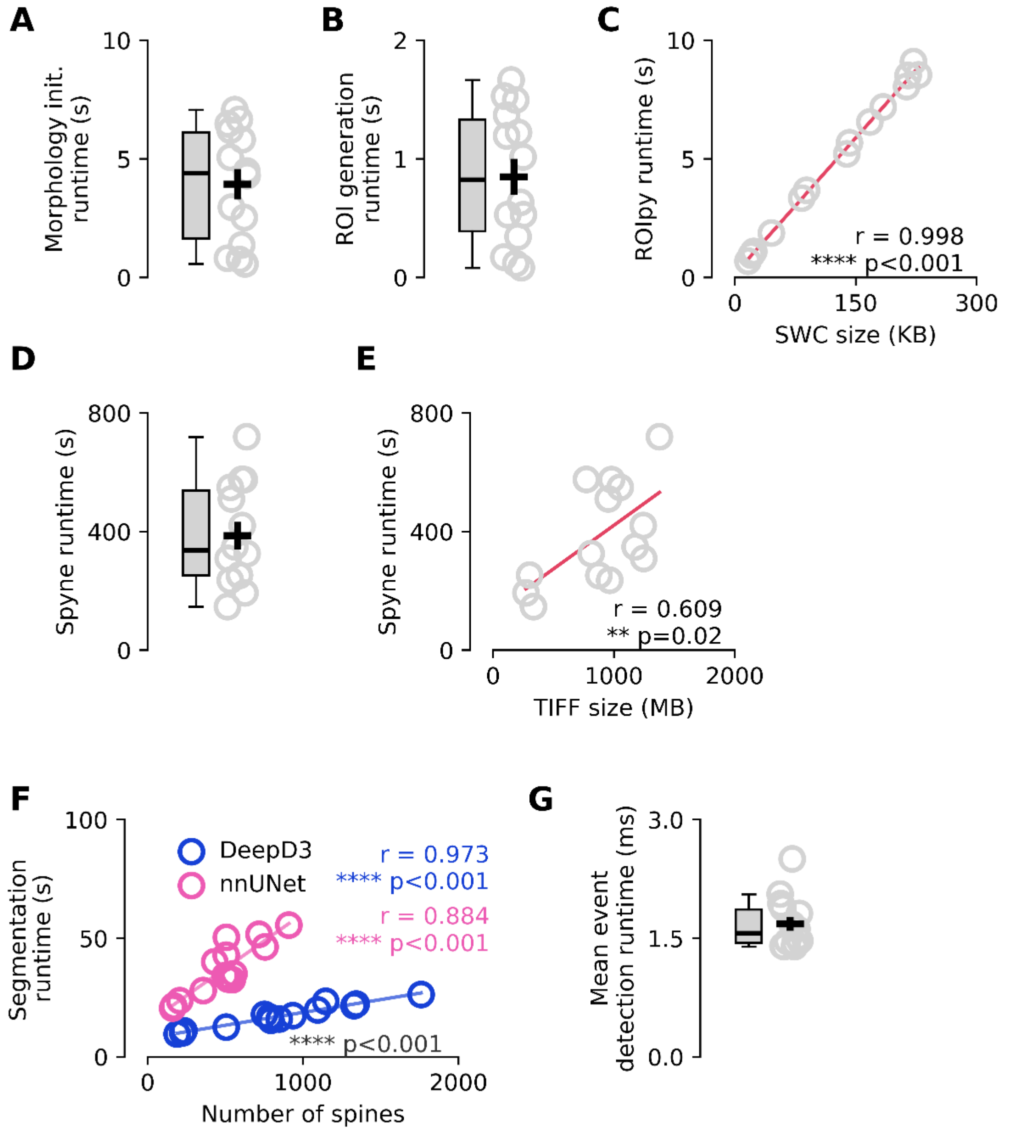

Figure S1. ROIpy and Spyne achieve efficient computational performance across image analysis workflows. Related to Figure 1, Figure 2 and Figure 3.

(A) Mean morphology instance initialization runtime (3.93 ± 0.64 s, n=14 neurons). Data are represented as mean +/- SEM. (B) Mean ROI generation runtime (0.85 ± 0.15 s, n=14 neurons). Data are represented as mean +/- SEM. (C) Correlation between ROIpy total processing time (morphology initialization + ROI generation) and SWC file size (Pearson r=0.99, p<0.001, n=14 neurons). SWC file size serves as a proxy for morphological complexity (number of nodes in the reconstruction). (D) Mean runtime for Spyne-based offline preprocessing of imaging datasets (386.37 ± 46.29, n=14 neurons). Data are represented as mean +/- SEM. (E) Correlation between total TIFF file size and pre-processing runtime (Pearson r=0.61, p=0.02, n=14). TIFF file size (MB) reflects imaging data volume. (F) Relationship between spine segmentation runtime and number of detected spines for DeepD3 versus nnUNet algorithms. DeepD3: 852.8 ± 123.9 spines, Pearson r=0.97, p<0.001, n=14 neurons; nnU-net: 488.5 ± 58.3 spines, Pearson r=0.88, p<0.001, n=14 neurons. Regression comparison (ANCOVA interaction, t=6.25, p<0.001).(G) Mean event detection runtime per individual trace using custom calcium event classifier (1.68 ± 0.09 ms, n=14 datasets). Data are represented as mean +/- SEM.

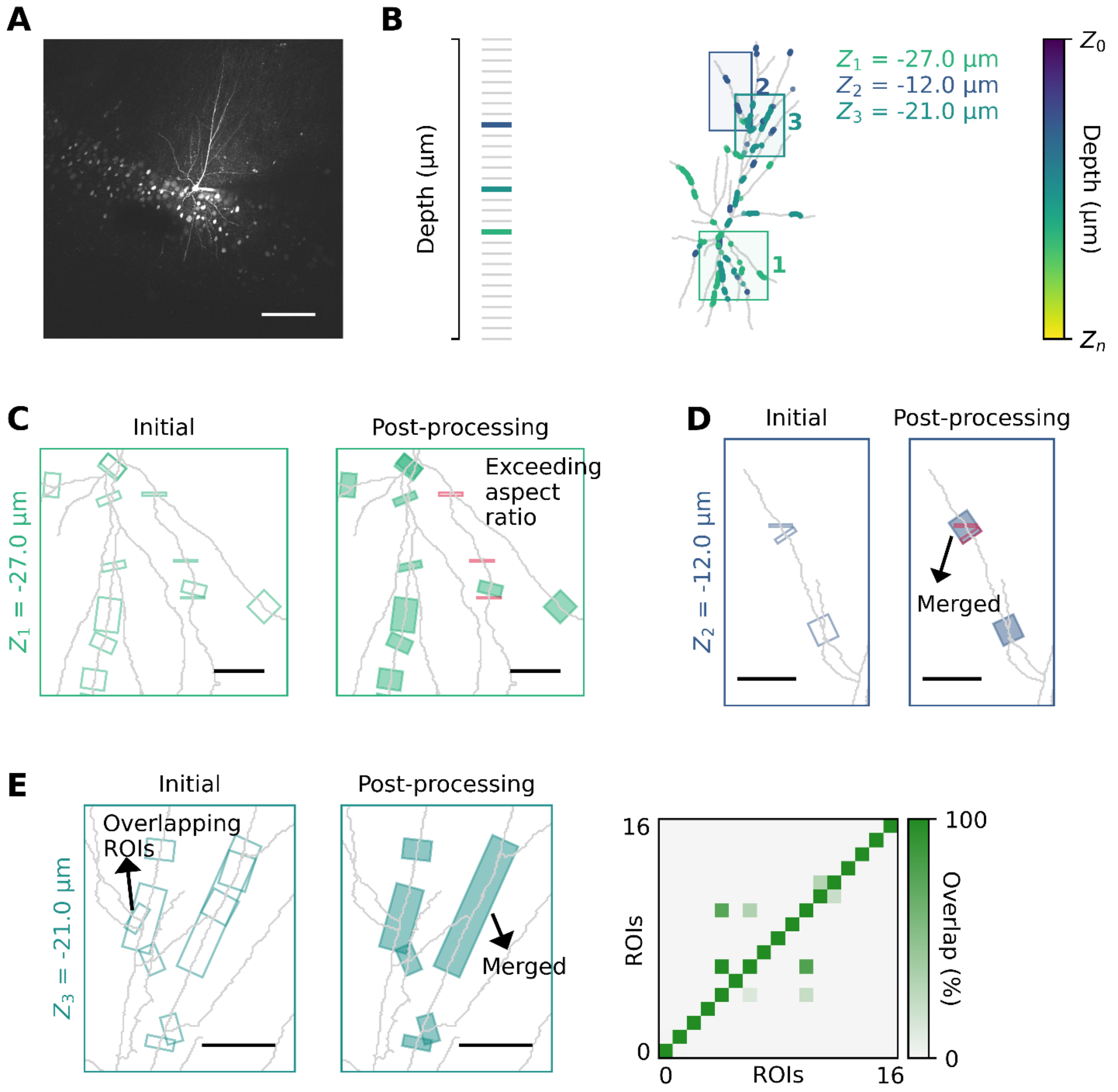

Figure S2. Automated ROI generation and post-processing workflow implemented in ROIpy.

(A) Left: maximum intensity projection of a representative image stack showing a patched CA1 PN filled with A594. Scale bar: 50 µm. (B) Schematics of three selected z-planes extracted from the stack shown in (A). Right: reconstructed neuronal backbone, with highlighted dendritic segments color-coded by the selected z-plane. Colored boxes indicate the dendritic regions shown in panels (C), (D) and (E). (C) Roi filtering based on aspect ratio. Left: initial ROIs placed on coplanar dendritic segments (blue). Right: retained ROIs after filtering (red); filtered ROIs exceeding aspect ratio threshold (blue). Scale bar: 20 µm. (D) Merging of adjacent and coplanar ROIs. Left: pre-processing placement of ROIs on dendritic segments. Right: post-processing showing merged ROIs. Scale bar: 20 µm. (E) ROI overlap filtering and merging. Left: example group with overlapping ROIs. Center: filtered and merged ROIs after processing; most overlapping ROI removed. Scale bar: 20 µm. Right: overlap matrix indicating the fraction of each ROI's area covered by another ROI.

A

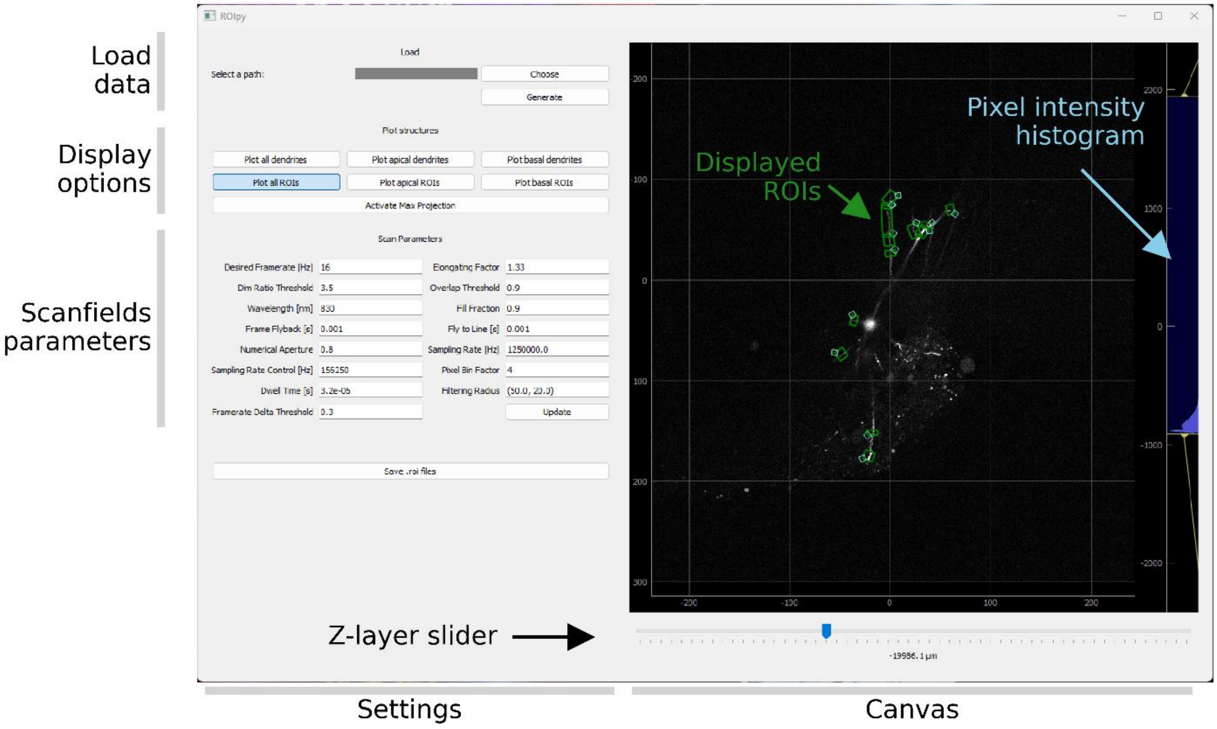

Figure S3. Graphical user interface supporting ROI generation and visualization in ROIpy. Related to Figure 1.

(A) The main layout consists of a setting panel and an interactive canvas. The settings panel includes options for loading data, interactive visualization controls such as toggling overlays, and adjusting ROI (scanfield) parameters. The canvas provides a z-layers navigation slider and includes a tunable pixel intensity histogram (blue). Placed ROIs (green) are displayed in reference to the underlying dendritic morphology.

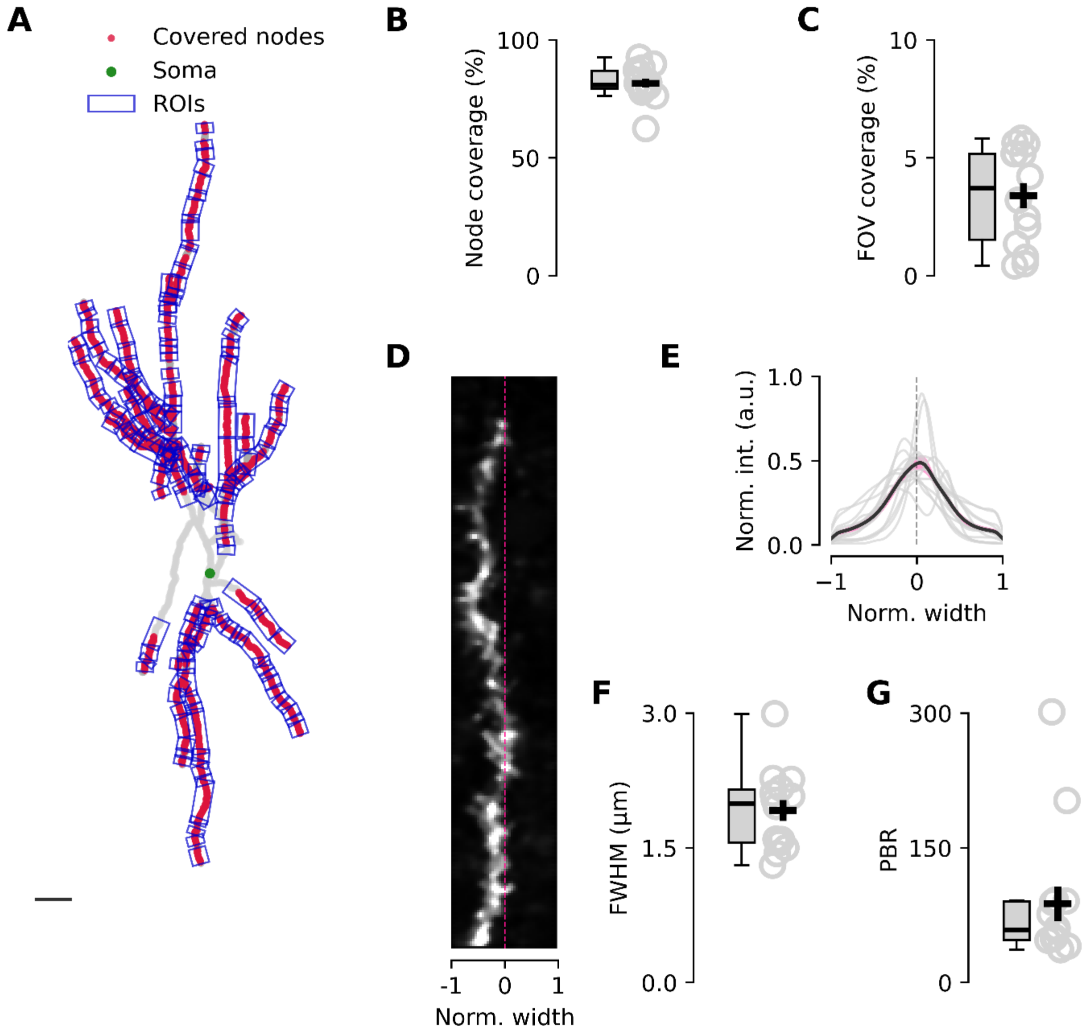

Figure S4. ROIpy accurately targets dendritic structures during automated ROI placement. Related to Figure 1.

(A) Representative xy projection of a reconstructed neuronal arbor with ROIs overlaid. Red dots indicate SWC nodes enclosed within ROIs; the green dot indicates the soma; blue rectangles indicate individual ROIs. Scale bar: 50 µm. (B) Percentage of SWC nodes enclosed by ROIs across neurons (81.61 ± 1.90%, n=14 neurons). Data are represented as mean +/- SEM. (C) Percentage of the total field-of-view area covered by all ROIs per neuron (3.39 ± 0.54%, n=14 neurons). Data are represented as mean +/- SEM. (D) Representative ROI image with the longitudinal midline marked (dashed pink line), used as reference for computing transverse fluorescence profiles orthogonal to the dendritic axis. By design, ROIs are placed with their longitudinal axis aligned with dendritic trajectory. (E) Transverse fluorescence intensity profiles across ROIs. Gray lines indicate the mean profile per neuron; black line indicates the mean across all neurons; shaded area indicates SEM. Fluorescence peaks at the ROI midline, confirming alignment of ROIs with the underlying dendritic segment. (F) Full width at half maximum (FWHM) of transverse fluorescence profiles (1.92 ± 0.12 µm, n=14 neurons), consistent with expected dendritic diameters and the optical resolution of the system. Data are represented as mean +/- SEM. (G) Peak-to-background ratio (PBR), defined as the ratio of fluorescence intensity at the ROI midline to that at the ROI periphery (87.71 ± 19.26, n=14 neurons) indicating high spatial specificity of dendritic targeting. Data are represented as mean +/- SEM.

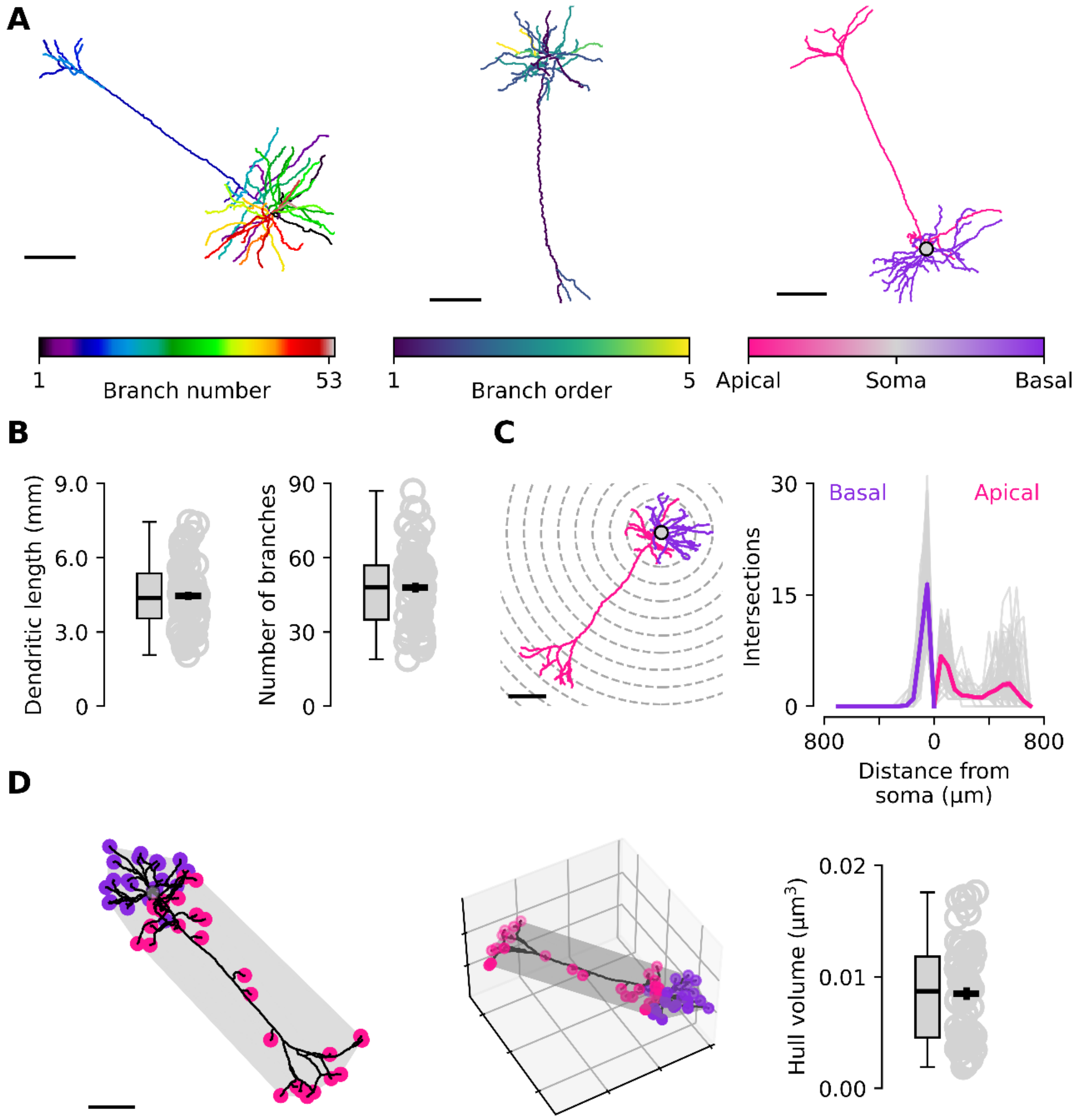

Figure S5. Morphological analysis of L5 pyramidal neurons using ROIpy.

(A) Representative morphological reconstruction of L5 PNs of the VISp, obtained from the Allen cell-type database and processed with *ROIpy*. Left: branches color-coded as individual segments from the soma or bifurcation points. Center: branches mapped by their branching order (*e.g.*, first order from soma, second order from first order, etc.). Right: branches grouped by dendritic compartment (apical vs. basal). Scale bars: 100 µm. (B) Left: mean total dendritic length (4461.38 ± 171.70 mm, n=63 neurons). Right: mean number of branches (47.89 ± 1.98, n=63 neurons). Data are represented as mean +/- SEM. (C) Sholl analysis. Left: schematic of concentric circles (50 µm step) centered on the soma to quantify dendritic complexity. Scale bar: 100 µm. Right: number of dendritic intersections with Sholl circles as a function of radial distance, plotted separately for apical (mean max. intersections: 6.73 ± 0.33, n=63 neurons) and basal (mean max. intersections: 16.41 ± 0.70, n=63 neurons) branches. Individual neurons are shown in gray; average profiles with SEM shading are shown in pink (apical) and violet (basal). (D) Convex hull volume analysis. Left: xy projection of the minimal convex hull enclosing dendritic endpoints. Scale bar: 100 µm. Center: 3D hull view with 100 µm grid spacing. Right: hull volume (0.0085 ± 0.0006 µm^3, n=63 neurons). Data are represented as mean +/- SEM.

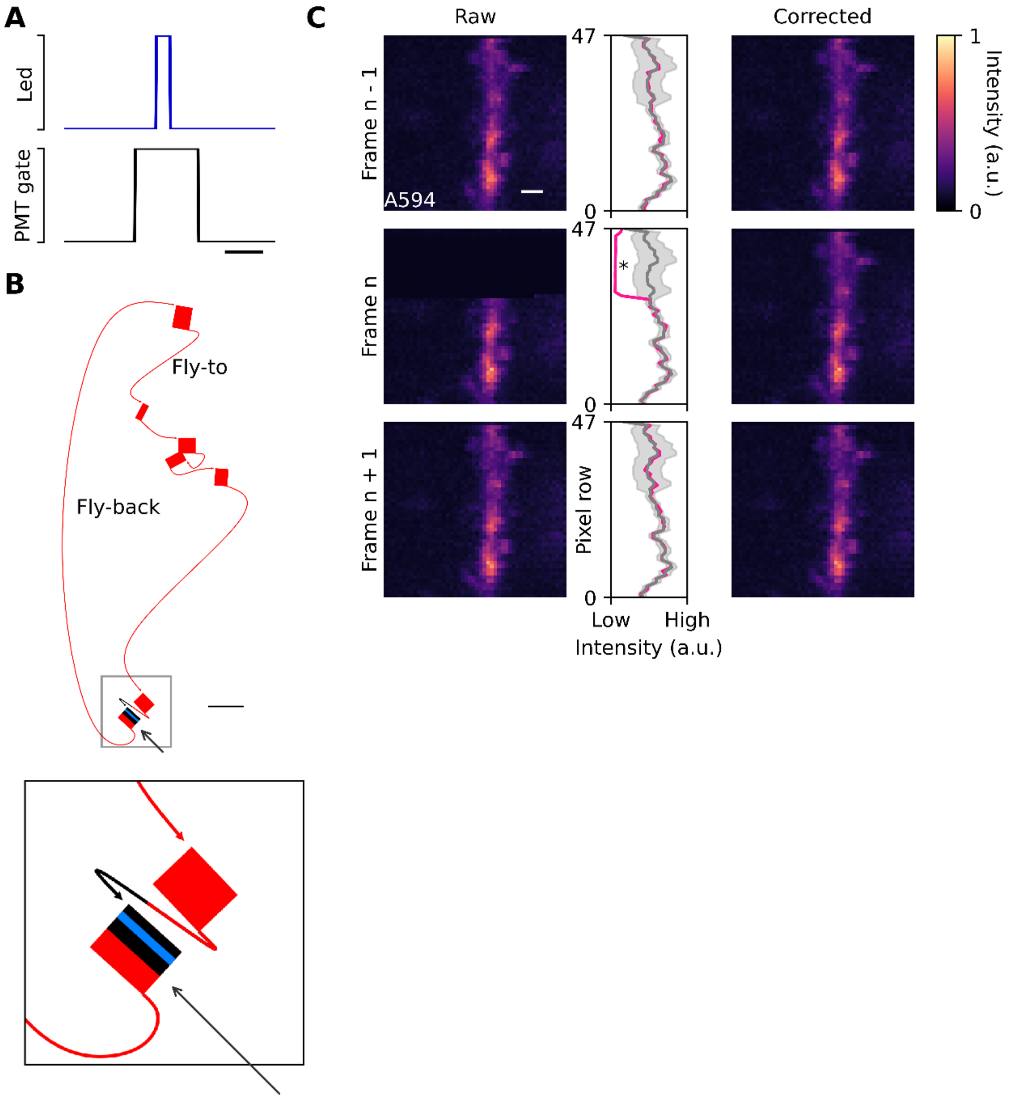

Figure S6. Detection and correction of PMT gating artifacts during optogenetic stimulation. Related to Figure 2C-D.

(A) Digital-to-analog square functions showing timing relationship between blue-light delivery (blue trace, top) and PMT gate (black trace, bottom). The PMT gate is triggered shortly before light onset and remains active for 10 ms to prevent photodamage. This results in a horizontal dark artifact in the image (shown in panel D). Scalebar: 5 ms. (B) Top: schematic of the scanner path and PMT gating during single z-layer imaging. Red rectangles indicate scanned sparse ROIs; curved arrows represent connecting travel (fly-to next ROI and fly-back to start position). Bottom: zoom-in of the PMT gate (black) activation just before scanning the ROI (as indicated by the partially black colored arrow). During this inactive period, blue light illuminates the sample (light blue line). Scale bar: 20 µm. (C) PMT gating artifact correction. Left column: raw frames from before (n – 1), during (n), and after (n + 1) stimulation showing a segment of A594-filled dendrite. Middle column: row-wise average intensity for each frame. Gray lines represent the mean row intensity across all 50 frames, with shaded areas indicating the tolerance threshold of 3σ. Pink traces correspond to the intensity profile of the current frame; rows exceeding the threshold are marked (*). Right column: corrected frame (n), where affected rows are interpolated using the corresponding rows from adjacent frames. Scale bar: 500 nm.

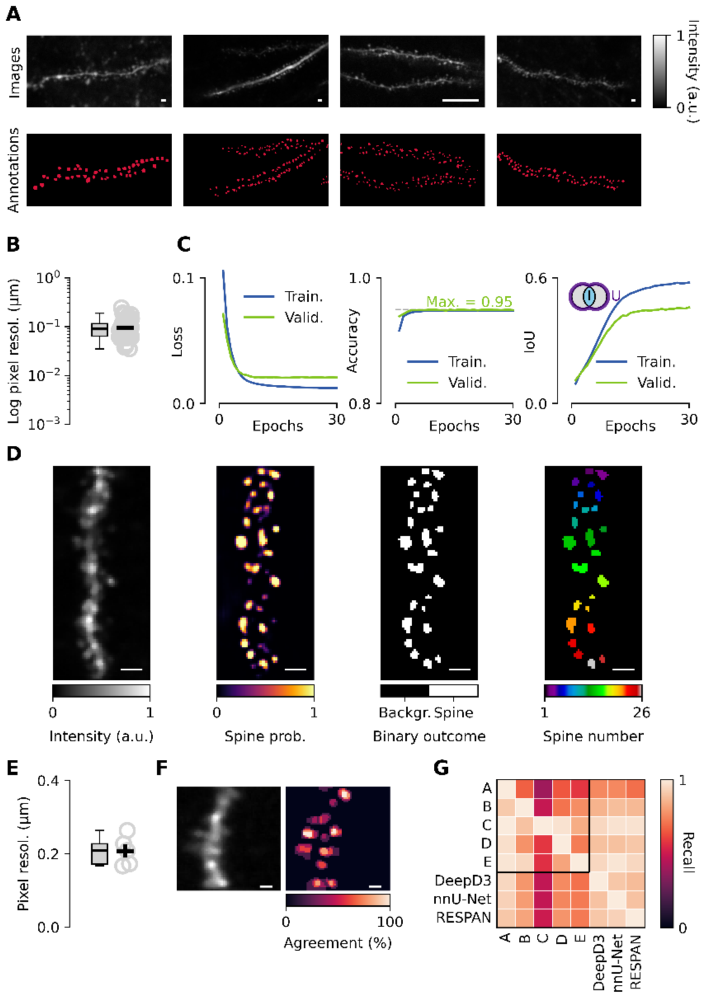

Figure S7 Spine segmentation model training and validation. Related to Figure 2B.

(A) Representative training image pairs. Top: four raw fluorescence images of dendritic segments drawn from the training dataset. Bottom: corresponding manual annotations with spine centroid locations marked in red, used as ground-truth labels. Scale bars: 2 µm. (B) Pixel resolution of training images (0.096 ± 0.006 µm; n = 50 images), reflecting the range of imaging conditions represented in the training set. Data are represented as mean +/- SEM. (C) Training and validation metrics. Left: binary cross-entropy loss. Middle: pixel-wise classification accuracy. Right: Intersection over Union (IoU) score. Training (blue) and validation (green) curves converge without divergence, indicating stable training without overfitting. (D) Spine segmentation pipeline on a representative ROI. From left to right: raw fluorescence image; per-pixel spine probability map output by the network; thresholded binary mask; individually labeled spine instances, color-coded by spine number. Scale bars: 2 µm. (E) Pixel resolution of images in the manual annotation validation dataset (0.21 ± 0.026 µm; n = 5 dendritic regions), used for benchmarking segmentation performance against human raters. Data are represented as mean +/- SEM. (F) Per-pixel rater agreement map for the representative ROI shown on the left. Color encodes the percentage of human raters classifying each pixel as spine (0% full disagreement, 100% full consensus). Scale bars: 2 µm. (G) Recall matrix comparing spine detection performance across five human raters (A–E) and three automated segmentation models (DeepD3, nnU-Net, RESPAN). Rows indicate the reference annotator; columns indicate the scorer. The black outline separates human raters (top-left block) from automated models (bottom rows and right columns). All three models achieve recall comparable to or exceeding mean inter-rater agreement.

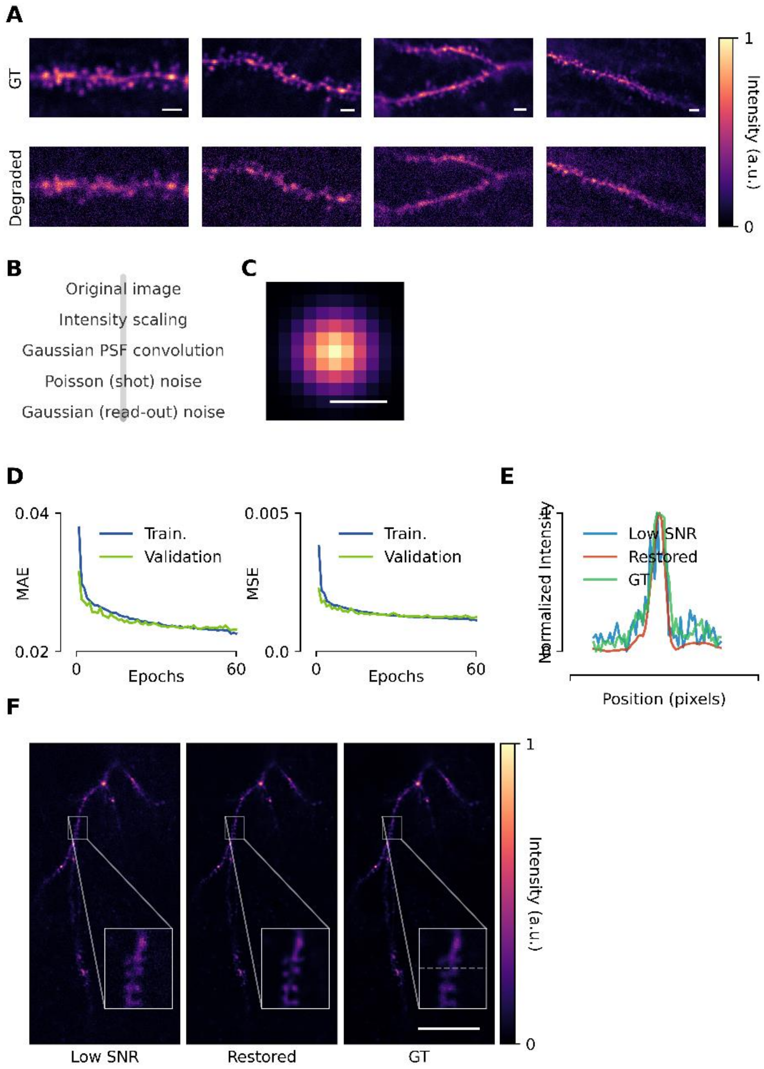

Figure S8. Synthetic image degradation pipeline and CSBDeep restoration model training.

(A) Example training image pairs. Top: four representative ground-truth fluorescence images of dendritic segments. Bottom: corresponding synthetically degraded images with reduced signal-to-noise ratio, used as model inputs during training. Scale bar: 2 μm. (B) Sequential steps of the synthetic degradation pipeline applied to each ground-truth image: intensity scaling, Gaussian PSF convolution, Poisson (shot) noise, and Gaussian (read-out) noise addition. (C) Example theoretical point spread function (PSF) used for Gaussian blurring during degradation. PSF width was scaled to pixel units individually per image to reflect variability in imaging conditions. Scale bar: 500 nm. (D) Training and validation loss curves across epochs. Left: mean absolute error (MAE), used as the primary training objective. Right: mean squared error (MSE), monitored as a secondary metric. Both metrics decrease and converge without divergence between training and validation sets, indicating stable model training. (E) Intensity profile along the line indicated in (F), drawn orthogonal to the dendritic shaft. The restored image recovers signal amplitude substantially closer to the ground truth relative to the low-SNR input, with marked reduction in background noise. (F) Representative test image. The same dendritic region was imaged under optimal laser power (right, ground truth, GT) and under reduced laser power (left, low SNR). The restored image (middle) was obtained by applying the trained CSBDeep model to the low-SNR input. Insets show magnified dendritic regions. The dashed line in the GT inset indicates the position of the intensity profile shown in (E). Scale bar: 10 μm.

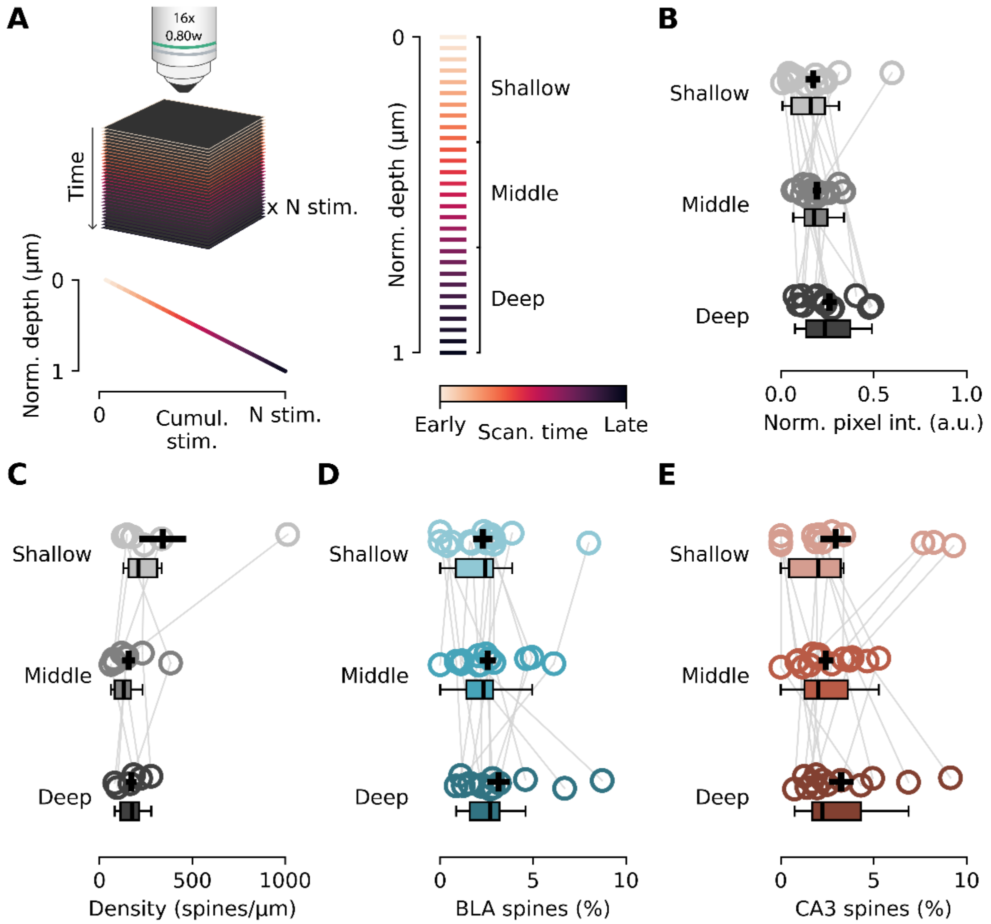

Figure S9. Depth-wise analysis of photobleaching, spine density, and spine responsiveness.

(A) Schematic illustrating the relationship between acquisition order and cumulative stimulation exposure. Top: during sequential z-layer acquisition, each layer is imaged for a fixed number of stimulus repetitions (N stim.), such that deeper layers are acquired later in the session and therefore accumulate greater total stimulation exposure (bottom left). To assess whether this cumulative exposure affected signal quality or spine responsiveness, z-layers were divided into three equal depth blocks — shallow, middle, and deep — color-coded by relative scan time from early (light) to late (dark) (right). (B) Normalized pixel intensity comparison across depth blocks (shallow: 0.17 ± 0.04; middle: 0.19 ± 0.02; deep: 0.26 ± 0.04; n=14 neurons No significant difference was detected across blocks (Kruskalis-Wallis: H=3.42, p=0.18; Dunn post-hoc with Bonferroni correction: shallow-deep p=0.19, middle-deep p=0.99). Data are represented as mean +/- SEM. (C) Mean spine density across depth blocks (shallow: 340.56 ± 125.88 spines/μm, middle: 156.18 ± 35.17 spines/μm, deep: 171.3 ± 27.75 spines/μm; n=14 neurons). Density did not differ significantly across blocks (Kruskalis-Wallis: H=3.33, p=0.19; Dunn post-hoc with Bonferroni correction: shallow-middle p=0.20, shallow-deep p=0.99). Data are represented as mean +/- SEM. (D) Fraction of BLA-responsive spines across depth blocks (shallow: 2.3 ± 0.5 %; middle: 2.6 ± 0.4 %; deep: 3.1 ± 0.6 %; n=14 neurons). No significant difference was found across blocks (Kruskal-Wallis: H=0.99, p=0.61; Dunn post-hoc with Bonferroni correction: all pairwise comparisons p ≥ 0.99). Data are represented as mean +/- SEM. (E) Fraction of CA3-responsive spines across depth blocks (shallow: 2.9 ± 0.8%; middle: 2.4 ± 0.4%; deep: 3.2 ± 0.7%; n=14 neurons). No significant difference was found across blocks (Kruskal-Wallis: H=0.42, p=0.81; Dunn post-hoc with Bonferroni correction: all pairwise comparisons p = 1.0). Data are represented as mean +/- SEM.

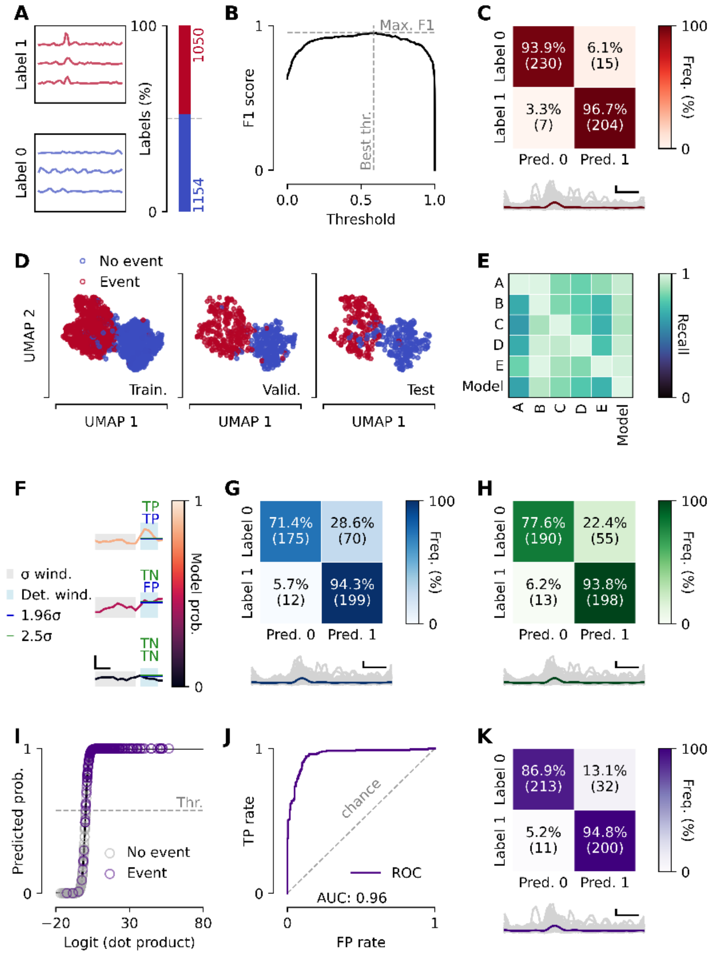

Figure S10. Classifier performance and benchmarking against classical detection methods. Related to Figure 3.

(A) Representative event (label 1, red; n = 1,050) and no-event (label 0, blue; n = 1,154) traces from the training dataset, with class proportions (bar plot). (B) F1 score vs. classification threshold on the held-out test set. Dashed line: optimal threshold (0.59). (C) Confusion matrix for the CNN at optimal threshold (sensitivity: 96.7%; specificity: 93.9%; accuracy: 95.2%). Below: mean correctly classified event trace (dark red) and individual traces (gray). Scale bars: 1 $\Delta F/F_0$, 500 ms. (D) UMAP of final-layer activations for training, validation, and test sets, color-coded by ground-truth label. Event and no-event form well-separated clusters across all splits. (E) Inter-rater recall matrix for five human annotators (A–E) and the model on the held-out test set. Model recall against individual raters: 0.91–0.97; human inter-rater range: 0.61–1.00. (F) Threshold-crossing detection logic. Traces exceeding 1.96σ or 2.5σ within the detection window (blue shade) are classified as events. Representative TP, TN and FP outcomes shown for both thresholds. Scale bars: 1 $\Delta F/F_0$, 200 ms. (G–H) Confusion matrices for threshold-crossing detection at 1.96σ (G; sensitivity: 94.3%; specificity: 71.4%; accuracy: 82.0%) and 2.5σ (H; sensitivity: 93.8%; specificity: 77.6%; accuracy: 85.1%). Scale bars: 1 $\Delta F/F_0$, 500 ms. (I) Logistic regression sigmoid mapping logit scores to event probability. Optimal threshold indicated (dashed line); traces color-coded by ground-truth label. (J) ROC curve for logistic regression on the held-out test set (AUC = 0.96). Dashed line: no-skill baseline. (K) Confusion matrix for logistic regression at optimal threshold (sensitivity: 94.8%; specificity: 86.9%; accuracy: 90.6%). Below: mean correctly classified events (dark violet) and individual traces (gray). Scale bars: 1 $\Delta F/F_0$, 500 ms.

Table S1. ROI generation parameters. Related to Figure 1, Figure S2, Figure S3 and Figure S4.

Parameter	Value	Notes
Initial ROI width	10 µm	Applied perpendicular to the dendrite axis
ROI elongation factor	1.33 × height	Applied along longitudinal axis
Aspect ratio threshold	3.5 (width/height)	Exclusion criterion based on aspect ratio
Overlap removal threshold	> 90% area overlap	Exclusion criterion to avoid redundant scanning
Soma exclusion radius	50 µm (apical), 20 µm (basal)	Exclusion criteria
Target frame rate	16 Hz	Defines the scan period (1 / frame rate)
Fly-to time (t_{fly-to}, per transition)	1 ms	Non-active scanning period between ROIs
Flyback time ($t_{flyback}$, per frame)	1 ms	Return time to the starting point of the frame scan
Fill fraction	0.9	Fraction of scanned area accounting for overscan
Sampling rate	1.250 MHz	ADC sample rate collection
Pixel bin factor	4	Number of ADC samples that are averaged into one pixel
Pixel dwell time ($tdwell$)	3.2 µs	Time to acquire a single pixel
Max iterations	100	Iterative loop limit to converge on target frame rate
Frame rate tolerance	± 0.3 Hz	Acceptable deviation from the target frame rate

Table S2. Dataset from Allen Cell-Types Database. Related to Figure S5.

Specimen ID	Donor Name	Cre Line	Structure	Hemisphere
485909730	205530	Cux2-CreERT2	VISp, L5	Right
323865917	172530	Scnn1a-Tg3-Cre	VISp, L5	Left
314804042	168053	Rorb-IRES2-Cre	VISp, L5	Left
488679042	212872	Rorb-IRES2-Cre	VISp, L5	Right
560678143	285657	Vip-IRES-Cre	VISp, L5	Right
471129934	180747	Rbp4-Cre_KL100	VISp, L5	Left
524850271	250843	Chrna2-Cre_OE25	VISp, L5	Left
486025194	205726	Rbp4-Cre_KL100	VISp, L5	Left
559387643	281934	Ctgf-T2A-dgCre	VISp, L5	Left
480114344	197332	Rorb-IRES2-Cre	VISp, L5	Right
589760138	318179	Sim1-Cre_KJ18	VISp, L5	Left
488697163	212873	Rorb-IRES2-Cre	VISp, L5	Right
485574721	203498	Rbp4-Cre_KL100	VISp, L5	Left
468120757	177297	Scnn1a-Tg3-Cre	VISp, L5	Right
515249852	243055	Rbp4-Cre_KL100	VISp, L5	Left
480122859	197352	Rorb-IRES2-Cre	VISp, L5	Left
502366405	230665	Ctgf-T2A-dgCre	VISp, L5	Left
486111903	205727	Rbp4-Cre_KL100	VISp, L5	Right

480116737	197332	Rorb-IRES2-Cre	VISp, L5	Right
488698341	212873	Rorb-IRES2-Cre	VISp, L5	Right
588402092	318850	Tlx3-Cre_PL56	VISp, L5	Right
480351780	197851	Rorb-IRES2-Cre	VISp, L5	Left
563226105	291923	Esr2-IRES2-Cre	VISp, L5	Left
607124114	338859	Tlx3-Cre_PL56	VISp, L5	Left
314822529	168053	Rorb-IRES2-Cre	VISp, L5	Left
471758398	180748	Rbp4-Cre_KL100	VISp, L5	Left
471767045	180748	Rbp4-Cre_KL100	VISp, L5	Right
471143169	180747	Rbp4-Cre_KL100	VISp, L5	Right
589442285	318531	Sim1-Cre_KJ18	VISp, L5	Left
473601979	181715	Rorb-IRES2-Cre	VISp, L5	Left
487664663	211531	Rbp4-Cre_KL100	VISp, L5	Left
476263004	187854	Scnn1a-Tg3-Cre	VISp, L5	Right
479225052	187416	Rorb-IRES2-Cre	VISp, L5	Left
480169178	197353	Rorb-IRES2-Cre	VISp, L5	Right
485911892	204780	Ntsr1-Cre_GN220	VISp, L5	Left
488695444	212873	Rorb-IRES2-Cre	VISp, L5	Left
485838981	204744	Rbp4-Cre_KL100	VISp, L5	Right

488680917	212872	Rorb-IRES2-Cre	VISp, L5	Right
479010903	195278	Pvalb-IRES-Cre	VISp, L5	Left
479225080	187416	Rorb-IRES2-Cre	VISp, L5	Left
485574832	203498	Rbp4-Cre_KL100	VISp, L5	Right
478586295	194322	Rorb-IRES2-Cre	VISp, L5	Right

Table S3. DeepD3 custom training parameters. Related to Figure 2 and Figure S7.

Parameter	Value	Description
Seed	41	Random seed for reproducibility
Filters	32	N of filters in the first convolutional layer
Batch size	32	Number of samples processed epoch
Learning rate	$5x10^{-4}$	Initial learning rate for Adam optimizer
Epochs	30	Number of complete passes through the training dataset
Patch size	128x128 pixels	Size of square patches sampled for analysis
Learning rate scheduler	Custom	Constant LR for epochs 1-15, then exponential decay by factor of $e^{-0.1}$ per epoch

Table S4. CARE custom training parameters. Related to Figure 2 and Figure S8.

Parameter	Value	Description
Axes	YX	Spatial axes of the input data (2D images)
N channel input	1	Number of input channels
N channel output	1	Number of output channels
U-net depth	2	Number of down/upsampling layers)
U-net kernel size	5	Kernel size for convolutional layers in the U-Net
U-net N features channel	32	Number of feature channels in the first U-Net layer
Epochs	60	Total number of training epochs
Batch size	16	Number of samples per training batch
Steps per epoch	400	Number of gradient update steps per epoch
Learning rate	$4x10^{-4}$	Initial learning rate for the optimizer
Learning rate drop factor	0.5	Factor by which learning rate is reduced on plateau
Learning rate drop patience	10	Number of epochs to wait before reducing learning rate

Table S5. Calcium event classifier training parameters. Related to Figure 3 and Figure S10.

Parameter	Value	Description
Input channel	1	Number of input channels
Conv. layer 1 channels	16	Number of output channels in first convolutional layer
Conv. layer 2 channels	32	Number of output channels in second convolutional layer
Conv. layer 3 channels	64	Number of output channels in third convolutional layer
Conv. layer 1 kernel size	5	Kernel size for first convolutional layer
Conv. layer 2 kernel size	3	Kernel size for second convolutional layer
Conv. layer 3 kernel size	2	Kernel size for third convolutional layer
Leaky ReLU negative slope	0.05	Negative slope coefficient for LeakyReLU activation
Dropout rate	0.1	Dropout probability for regularization
Pool layer kernel size	2	Kernel size for max. pooling layers
Epochs	500	Maximum number of training iterations
Learning rate	$1x10^{-5}$	Initial learning rate for optimizer
Learning rate drop factor	0.5	Factor by which learning rate is reduced on plateau
Learning rate drop patience	5	Number of epochs to wait before reducing learning rate
Lambda 1	$1x10^{-5}$	L1 regularization penalty coefficient
Lambda 2	$10x10^{-4}$	L2 regularization penalty coefficient
Patience	8	Early stopping (epochs without improvement)
Batch size	16	Number of samples per training batch
